# Natural Variation at *sympathy for the ligule* Controls Penetrance of the Semidominant *Liguleless narrow-R* Mutation in *Zea mays*

**DOI:** 10.1534/g3.114.014183

**Published:** 2014-10-24

**Authors:** Elizabeth M. Buescher, Jihyun Moon, Anne Runkel, Sarah Hake, Brian P. Dilkes

**Affiliations:** * Department of Horticulture and Landscape Architecture, Purdue University, West Lafayette, Indiana 47907; † Plant Gene Expression Center, USDA-ARS, University of California, Berkeley, Albany, California 94710

**Keywords:** genetic network, development, QTL, epistasis

## Abstract

Leaf architecture determines plant structural integrity, light harvesting, and economic considerations such as plant density. Ligules, junctions at the leaf sheath and blade in grasses, protect stalks from environmental stresses and, in conjunction with auricles, controls leaf angle. Previous studies in mutants have recessive liguleless mutants (*lg1* and *lg2*) and dominant mutations in *knotted1*-like homeobox genes (*Lg3-O*, *Lg4*, and *Kn1*) involved in ligule development. Recently, a new semidominant liguleless mutant, *Liguleless narrow* (*Lgn-R*), has been characterized in maize that affects ligule and auricle development and results in a narrow leaf phenotype. We show that quantitative genetic variation affects penetrance of *Lgn-R*. To examine the genetic architecture underlying *Lgn-R* expressivity, crosses between *Lgn-R*/+ mutants in a B73 background and intermated B73 x Mo17 recombinant inbred lines were evaluated in multiple years and locations. A single main-effect quantitative trait locus (QTL) on chromosome 1 (*sympathy for the ligule*; *sol*) was discovered with a Mo17-contributed allele that suppressed *Lgn-R* mutant phenotypes. This QTL has a genetic-interaction with a locus on chromosome 7 (*lucifer*; *lcf*) for which the B73-contributed allele increases the ability of the *sol^Mo17^* allele to suppress *Lgn-R*. Neither of the genetic intervals likely to contain *sol* or *lcf* overlap with any current liguleless genes nor with previously identified genome-wide association QTL connected to leaf architecture. Analysis of phenotypes across environments further identified a genotype by enviroment interaction determining the strength of the *sol x lcf* interaction.

The ligule is an important architectural feature of grass leaves that protects the stalk from disease and environmental insults. In maize, the ligule and auricles allow the leaf blade to lean away from the sheath, which remains wrapped around the stalk (Foster and Timmermans 2009; Kiesselbach 1949). Study of liguleless mutants has identified genes with roles in meristem maintenance, leaf polarity, and leaf angle (Moreno *et al.* 1997; Walsh *et al.* 1998). The latter is a trait of critical importance to plant density and maize agriculture (Duvick 2005). In maize and other grass species, mutant studies, and quantitative trait loci (QTL) mapping have identifed the genomic locations of loci affecting leaf architecture (Mickelson *et al.* 2002; Tian *et al.* 2011; Yu *et al.* 2012; Zeng *et al.* 2009).

Classical and contemporary mutant studies have identified genes required for ligule development: *lg1* is located on the short arm of chromosome 2; and *lg2* is located on the long arm of chromosome 3 (Beckett 1975). Maize homozygous for recessive loss-of-function alleles at *liguleless1* or *liguleless2* lack a ligule and auricles (Becraft and Freeling 1991; Harper and Freeling 1996; Moreno *et al.* 1997), resulting in more erect leaves. *lg1* encodes a protein similar to SQUAMOSA PROMOTER-BINDING (Moreno *et al.* 1997) and *lg2* encodes a basic leucine zipper protein transcription factor (Walsh *et al.* 1998). In addition to these, *lg3*, located on 3S (Kerstetter *et al.* 1994), and *lg4*, located on 8L (Fowler and Freeling 1991), were identified from dominant mutations that altered ligule formation. The semidominant *Liguleless3* alleles cause the leaf blade, ligule, and auricle tissues to adopt a sheath-like phenotype (Fowler and Freeling 1996). The *Liguleless4* dominant mutants, much like *Liguleless3*, show no auricle or ligule tissue, with blade tissue appearing to be sheath-like; however, the tissue disruption occurs near the leaf margins (Fowler and Freeling 1991). *liguleless3* and *liguleless4* encode class-I *knotted1*-like homeobox (KNOX) transcription factors that are duplicated within the grasses (Bauer *et al.* 2004; Kerstetter *et al.* 1994; Muehlbauer *et al.* 1999). The *knotted1* transcription factor (Vollbrecht *et al.* 1991) is also involved in ligule development as well as affecting shoot meristem development (Kerstetter *et al.* 1994; Volbrecht *et al.* 2000). *Kn1*-dominant mutants displace the position of the ligule into the blade and cause knot structures composed of mislocalized sheath tissue to form along the lateral veins (Sinha and Hake 1990; Vollbrecht *et al.* 1991).

Leaf architecture features involving the ligule and the leaf angle have been examined using QTL studies across several species (Mickelson *et al.* 2002; Tian *et al.* 2011; Yu *et al.* 2012; Zeng *et al.* 2009). In rice, five flag-leaf ligule length QTL were mapped to chromosomes 2, 6, 10, and 12 as well as the rice ortholog of maize *liguleless1* on the long arm of chromosome 4 (Zeng *et al.* 2009). Similarly, Tian *et al.* (2011) performed a large-scale QTL study in maize and found relatively large-effect QTL controlling leaf angle mapped to the *lg1* and *lg2* genes.

Recently, a semidominant mutant was identified that defines a novel locus affecting ligule development. *Liguleless-narrow* (*Lgn-R*) was identified in an ethane methyl sulfonate−mutagenized B73 population (Moon *et al.* 2013). *Lgn-R* plants exhibit disrupted ligule development and reduced auricles toward the margins of the leaf. The leaves are both shorter and narrower. In addition, *Lgn-R* mutants produce fewer tassel branches and often fail to produce an ear. Expression of the *Lgn-R* mutant phenotype is clear in B73 as well as other inbred backgrounds (Moon *et al.* 2013).

In this work, we describe variation in the penetrance of *Lgn-R* affected by natural genetic variation. *Lgn-R* phenotypic expression is suppressed in crosses to Mo17. To map the *Lgn-R* modifiers, length and width measurements were made on *liguleless-narrow* mutants from *Lgn-R*/+ individuals in the B73 background crossed to Intermated B73 x Mo17 (IBM; Beavis *et al.* 1992; Lee *et al.* 2002) recombinant inbred lines (RILs). The IBM RIL x *Lgn-R/*+ F1 were grown in two locations in 2009, Indiana (Purdue Agronomy Center for Research and Education; West Lafayette, IN) and California (Gill Tract Farm; Albany, CA). A single main-effect QTL was identified on chromosome 1, called *sympathy for the ligule* (*sol*). The Mo17 allele at *sol* suppresses mutant phenotype expression in *Lgn-R* plants resulting in increased fertility, leaf length, and leaf width. The RIL genotypes of fertile, successfully suppressed IBM RIL x *Lgn-R/*+ F1 individuals were analyzed for shared regions of homozygosity, which, along with genome-wide pairwise analysis for epistasis, identified an epistatic interaction between *sol* and a region of chromosome 7, referred to as *lucifer (lcf)*. Genotype by environment analysis revealed a dramatic requirement of environment for the expression of the *sol* x *lcf* interaction. We propose that this genetic network helps to integrate plant growth and architecture with environmental determinants of development.

## Material and Methods

### Phenotypic measurements

The dominant *Lgn-R* mutant was isolated as a half-plant chimera from ethane methyl sulfonate mutagenesis of the inbred line B73 (Gerhold *et al.* 2005; Moon *et al.* 2013). *Lgn-R*/+ plants from introgression of the allele into the Mo17 and B73 backgrounds were used in this study. To determine the existence and location of *Lgn-R* suppressors, B73 *Lgn-R* heterozygotes were crossed as the pollen donor to 63 individuals from the core set of 94 IBM (Beavis *et al.* 1992) thus creating RIL x *Lgn-R/*+ F1s. Leaf length and width measurement of the 6th leaf from the tassel were recorded for between two and eight individual F1 (IBM RIL x *Lgn-R*) plants from these crosses grown at the Purdue Agronomy Center for Research and Education (West Lafayette, IN) and University of California Gill Tract farm (Albany, CA) in 2009. In both locations, *Lgn-R*/+ individuals were distinguishable from wild-type siblings due to dramatically narrower and shorter leaves. From these measurements, leaf area (LA, length x width) and length by width (LW, the ratio of length to width) were calculated. The location in Indiana consisted of two independent plantings, and measurements and was thus treated as replicates: IN_1 and IN_2. In addition to leaf phenotypes, stand counts of plants in the row (count) were also made. In 2010, a subset of the IBM RIL x *Lgn-R*/+ F1 individuals (IBM18, IBM30, IBM69, and IBM72), which previously displayed rescued or near wild-type sibling leaf phenotypes, were grown at the Purdue Agronomy Center for Research and Extension (West Lafayette, IN) to confirm action of the *Lgn-R* modifiers. An additional five RIL (IBM4, IBM25, IBM47, IBM55, and IBM65) were identified as substantially rescued to wild-type phenotypes among the RIL x *Lgn-R* F1s grown for the 2009 mapping experiment. These nine individuals are referred to as the “rescuing RILs.”

### Data processing and QTL analysis

Within and between years and locations, all leaf traits were positively correlated within individuals and between replicates of each IBM RIL x *Lgn-R*/+ F1 family. To better describe the genetic effects on phenotype expression and capture the genetic covariance responsible for trait correlation, principal components analysis (PCA) of covariances between traits was performed using the JMP software package (version 8.0.1, SAS Institute Inc., 2009) to derive additional quantitative traits for QTL mapping. All traits (*e.g.*, length, width, count) were used for QTL mapping as well as the additional PCA derivatives presented in detail here: LW_all (length by width, all locations), LW_IN (length by width, IN locations), LW_GT (length by width, CA only), LA_all (leaf area, all locations), and LA_IN (leaf area, IN locations). Each IBM RIL had multiple length and width measurements and all individuals were used for PCA imputation. For comparisons between the IN and CA environments, leaf area and not principal components from CA was compared to leaf area from IN.

QTL analysis for all traits was conducted using R/qtl (Broman *et al.* 2003) using the 2009 data sets. Genotypes and map positions were based on the ISU IBM Map version 4 (http://www.maizeGDB.org) for the core 94 IBM population. Markers with more than 15% missing data among the 63 phenotyped IBM RIL were removed, leaving 3205 markers across the 10 maize chromosomes. Missing genotypes were imputed by the no double crossovers method, in which missing genotypes are filled in unless a recombination event is predicted to occur (R/qtl package manual; Broman *et al.* 2010). One-dimensional genome scans were performed using *scanone* by the Expectation Maximization method. *Scanone*, using method Expectation Maximization, assumes a single QTL model and follows standard interval mapping (R/qtl package manual; Broman *et al.* 2010). Experiment-wide permutation thresholds (alpha < 0.05) were determined separately for each trait with 1000 permutations. Two-dimensional genome scan and tests for epistasis were performed by marker-pair regression analysis using the *scantwo* function (Broman *et al.* 2003). *Scantwo* tests a two-QTL model and result summaries provide multiple assessments of interaction. *Scantwo* results were summarized using the “int” flag for interaction summary and by the “best” method to look for additional QTL by two dimensional scanning. Experiment-wide significance for the two dimensional scans (alpha < 0.05) were determined for each trait and procedure (“int” and “best”) with 1000 permutations. The complete mapping procedure, scripts, primary data, and PCA outputs are all available as a supplement to this mansucript or from the authors by request (Supporting Information, File S1).

A Standard Least Squares model implemented in the JMP statistical software package (version 8.0.1; SAS Institute Inc. 2009) examined the genotype by environment interactions for data from 2009 from both locations. The genotypes of both *sol*, estimated with marker umc2145, and *lcf*, estimated with marker nbp1, were compared with the leaf area traits for each location and across both locations.

### Validation of *sol* and *lcf* effect

At the Gill Tract farm in 2011, a subset of IBM RIL x *Lgn-R/+* F1 plants (IBM18, IBM30, IBM69, and IBM72) as well as BC1 IBM RIL x *Lgn-R* (IBM RIL x *Lgn-R* F1 backcrossed to B73) families segregating for the B73 and Mo17 alleles at *sol* were measured for leaf length and width of the 6th leaf from the tassel as well as plant height. A total of 187 individuals were measured. DNA was isolated from leaf samples for each individual and genotyped using the polymerase chain reaction markers umc2145 at chromosome one 94 cM for *sol* and bnlg1792 at chromosome seven 66 cM for *lcf*. Individuals were scored as either “B73,” “Mo17,” or heterozygous (B73/Mo17) for each marker. Because the RILs that effectively rescued *Lgn-R* plants were fixed for *lcf*^B73^ and the recurrent parent was B73, these BC1 plants were not segregating for *lcf*. To examine the effect of *sol* on mutant and wild-type leaf development, a comparison of leaf length and width data as well as plant height and the *sol* genotype was estimated by a Standard Least Squares model using the JMP software package (version 8.0.1, SAS Institute Inc. 2009).

## Results

**Figure 1:**
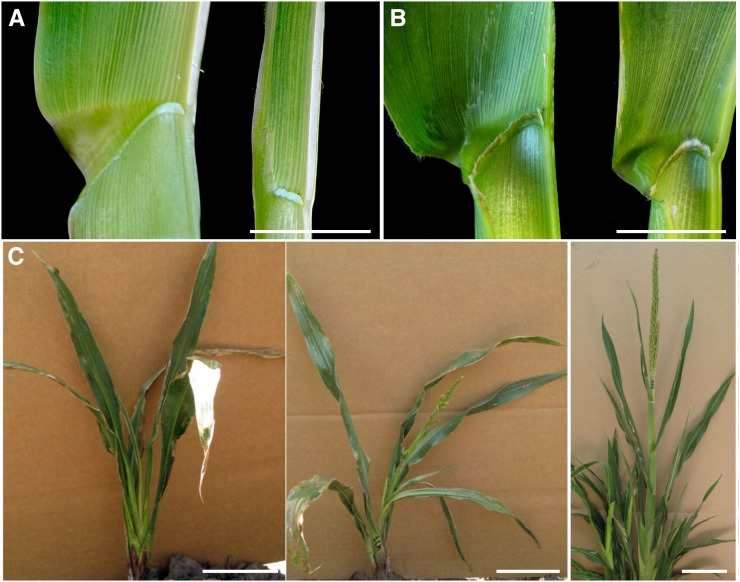
The effect of the Mo17 background on the *Lgn-R*/+ phenotype. (A) Ligular region of wild-type (left) and *Lgn-R*/+ (right) in B73 grown in Albany, CA. (B) Ligular region of wild type (left) and *Lgn-R*/+ (right) in Mo17 in West Lafayette, IN. (C) Whole plants grown in West Lafayette, IN. *Lgn-R*/+ in B73 (left) is compared to rescued *Lgn-R*/+ F1s (center and right) generated by crosses to IBM lines. Scale bars: 10 cm.

The dominant *Lgn-R* mutation results in narrower and shorter leaves, reduced ligule, fewer leaves, failure of ear development, and reduced tassel branches compared with recessive wild-type alleles (Figure 1). Introgression of *Lgn-R* into the Mo17 background partially suppresses the mutant phenotype (Table 1). These observations suggest the presence of genetic modifiers of *Lgn-R* that are polymorphic between Mo17 and B73. To identify and localize modifying loci, we used the intercrossed RIL population developed from a B73 x Mo17 cross (referred to as IBM; Beavis *et al.* 1992; Lee *et al.* 2002). Crosses were made using *Lgn-R* pollen in the B73 background onto IBM lines to give rise to IBM RIL x *Lgn-R/+* F1s. Stand counts, leaf number, leaf length, and leaf width measurements were made on the 6th leaf from the tassel of each F1 indivdual grown in 2 locations: Gill Tract, CA, and West Lafayette, IN, in the summer of 2009.

**Table 1:** “Rescuing” RIL x *Lgn-R* F1 lines and the Mo17 parent leaf morphology traits from both the IN and CA 2009 data

*Lgn-R* F1 Genotype*^a^*	Location*^b^*	Length*^c^*	Width	Leaf Area	LbW
RIL x *Lgn-R*/+ (IBM4)	IN1	54.00*^d^*	5.17	279.25	10.44
	IN2	56.13	4.35	244.14	12.90
	CA	69.79	8.50	593.18	8.21
RIL x *Lgn-R*/+ (IBM25)	IN1	56.14	3.97	222.96	14.14
	IN2	56.77	3.74	212.60	15.16
	CA	81.29	8.80	715.31	9.23
RIL x *Lgn-R*/+ (IBM47)	IN1	55.63	4.09	227.37	13.61
	IN2	54.43	3.77	205.27	14.43
	CA	79.75	8.65	689.84	9.21
RIL x *Lgn-R*/+ (IBM55)	IN1	46.33	3.42	158.56	13.53
	IN2	48.33	3.51	169.70	13.77
	CA	70.27	7.42	521.38	9.46
RIL x *Lgn-R*/+ (IBM65)	IN1	48.88	3.59	175.33	13.62
	IN2	51.00	3.58	182.58	14.24
	CA	71.42	7.38	526.70	9.68
Mo17 (Mo17 x *Lgn- R*/+)	IL1	59.67	4.38	261.53	13.61
	IL2	62.00	4.43	274.86	13.89
	CA	82.35	10.61	873.91	7.76
Wild-type Mo17	IN1	91.40	11.30	1032.82	8.09
	IN2	87.40	11.22	980.63	7.79
	CA	97.60	12.32	1202.43	7.92

RIL, recombinant inbred line; QTL, quantitative trait loci.

a RIL x *Lgn-R* F1 genotypes were identified as partially rescued phenotypes by the 2009 IBM RIL QTL mapping data.

b Three locations are listed, two measurements made in West Lafayette, IN, and one made in Gill Tract, CA.

c All phenotype measurements were made on the 6th leaf from the tassel. Length and width measurements are the mean value for four to eight individuals and leaf area (length × width) and LbW (length by width) measurements were calculated using length and width measurements.

d All measurements are in cm units.

**Figure 2:**
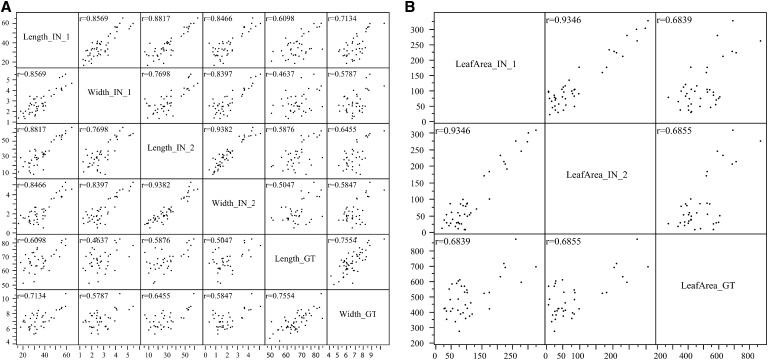
Correlation matrices for principal component analysis values for two traits, length and width across all locations (A) and (B) leaf area across location. Red ellipse indicates α = 0.05.

Leaf length and width measurements were highly correlated across environments within and across locations (Figure 2). Correlation of length and width traits for location GT (CA) had an r = 0.7397 (Figure 2A). Both length and width measurement correlations in location IN and location GT were statistically significant at *P* < 0.001 (data not shown). We calculated two other traits by using the leaf length and width measurements: leaf area (length × width) and a length width ratio (length:width). The calculated leaf area for each individual was also highly correlated across environments (r = 0.6839 and *P* < 0.001) as well as within location IN (r = 0.9346 and *P* < 0.001) (Figure 2B). The correlation of leaf measurements allowed a PCA to generate single values that better represented the genetic effect on leaf development of each IBM RIL. Thus, use of the PCA should increase both the sensitivity and accuracy of QTL mapping. To create values for the multiple traits measured, PCA of covariances defined a single trait for length and width measurements across both locations (LW_all) as well as locations separately (LW_IN and LW_GT); and finally, leaf area across all locations (LA_all) and leaf area in IN only (LA_IN). Principal component values for LW_all, LW_IN, LW_GT, LA_all, and LA_IN explained much of the variation in the measured traits (87.06%, 94.08%, 99.01%, 83.83%, and 96.74%, respectively).

### QTL mapping

**Figure 3:**
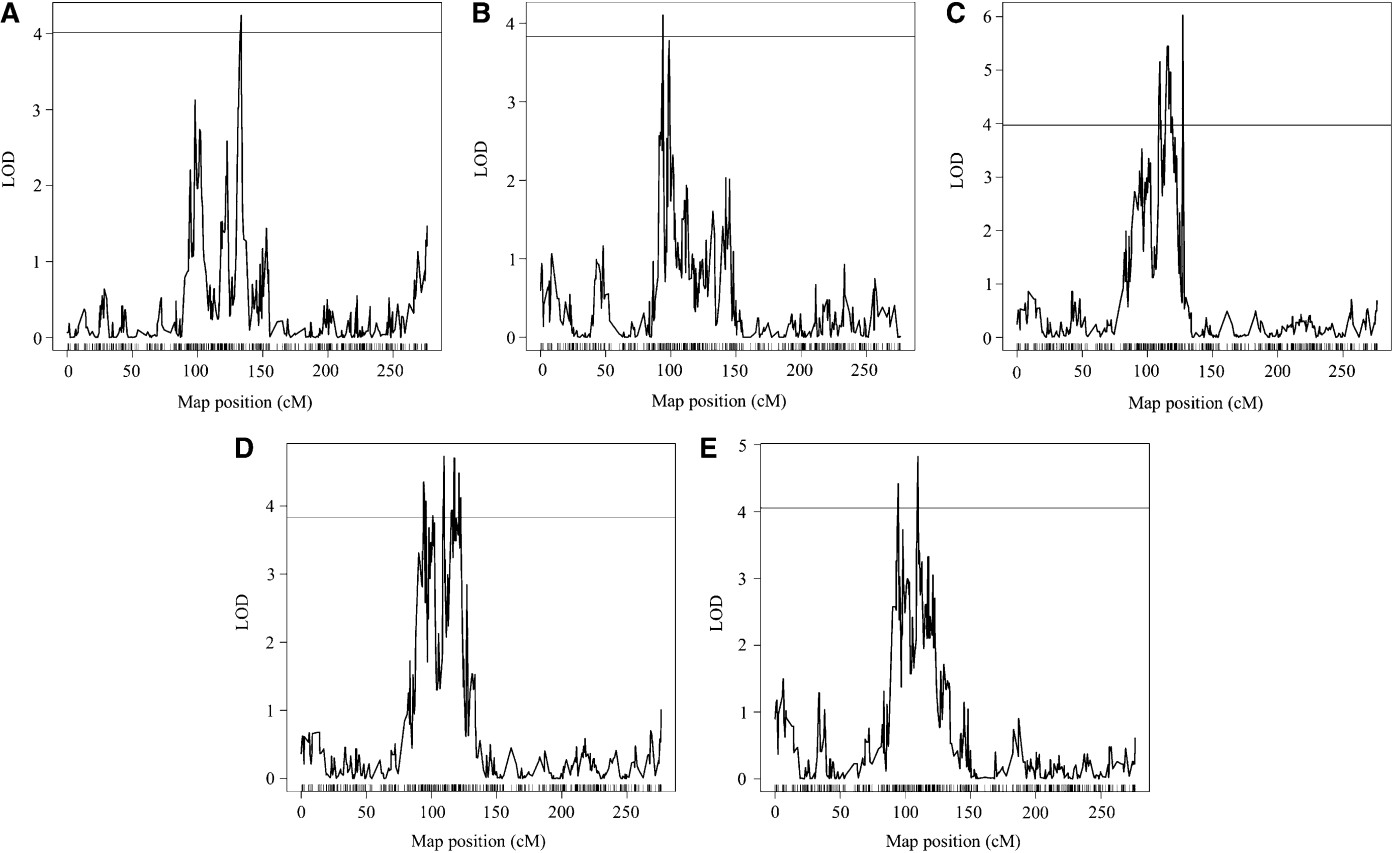
Interval mapping QTL results for leaf morphology traits. All figures show the LOD score on the x-axis and map position for chromosome 1 only on the y-axis. An experiment-wide permutation threshold was calculated for each trait and the α = 0.05 threshold is represented by the line on each figure. (A) Count, location IN2 trait. (B) Leaf Area, location CA trait. (C) Leaf area, location IN trait. (D) Location IN trait. (E) Length and width for all locations trait.

One-dimensional genome scans identified a single QTL, *sympathy for the ligule* (*sol*), on the short arm of chromosome 1 (Table 2). Multiple traits shared this QTL, and it was detected with: count, location IN2 (count_IN2); leaf area, location CA (LA_GT); leaf area, location IN (LA_IN); location IN (length and width PCA value; LW_IN); and length and width for all locations (LW_all) (Figure 3 and Table 2). Localization of *sol* by a two LOD drop-off for the QTL affecting LW_all mapped *sol* to likely be between 92.9 and 121.6 cM on chromosome 1 in the IBM ISU v4 map. A 95% Bayesian credible interval was substantially less specific, estimating *sol* to be between 5.6 cM and 133.9 cM on chromosome 1.

**Table 2:** Interval mapping QTL summary for each trait

	LOD score*^a^*	Marker*^b^*	Chr	Position	CI.low*^c^*	CI.high	5% LOD*^d^*
LA_GT	4.1*	umc2145	1	94	91.7	99.4	3.66
LW_GT	−	−	−	−	−	−	3.64
Count GT	−	−	−	−	−	−	4.01
Count IN1	−	−	−	−	−	−	4.04
Count IN2	4.242*	bnl5.59a	1	133.5	97.5	135	4.11
LA_IN	6.02*	IDP1423	1	27.1	109	128	4.27
LW_IN	4.726*	umc2229	1	109.6			3.90
LW_all	4.826*	umc2229	1	109.6	93.4	111	4.08
LA_all	−	−	−	−	−	−	−

QTL, quantitative trait loci; LOD, xxx.

a The LOD score is indicated if a QTL was detected for that trait and noted with a * if the LOD score is above the experiment-wide permutation threshold at α = 0.05.

b Markers, chr (chromosome) and position are from the Illinois State University IBM map (version 4, http://www.maizegdb.org).

c Confidence intervals (CI.low and CI.high) are indicated for each QTL, with lower CI indicated as CI.low and the higher CI indicated as CI.high.

d The experiment-wide permutation threshold at α = 0.05 for each QTL, as calculated using R/qtl (see the section *Materials and Methods*).

* Denotes statistical significance at α = 0.05.

### Validation of extreme rescue phenotypes in IBM x *Lgn-R* detects the *lcf* locus

**Figure 4:**
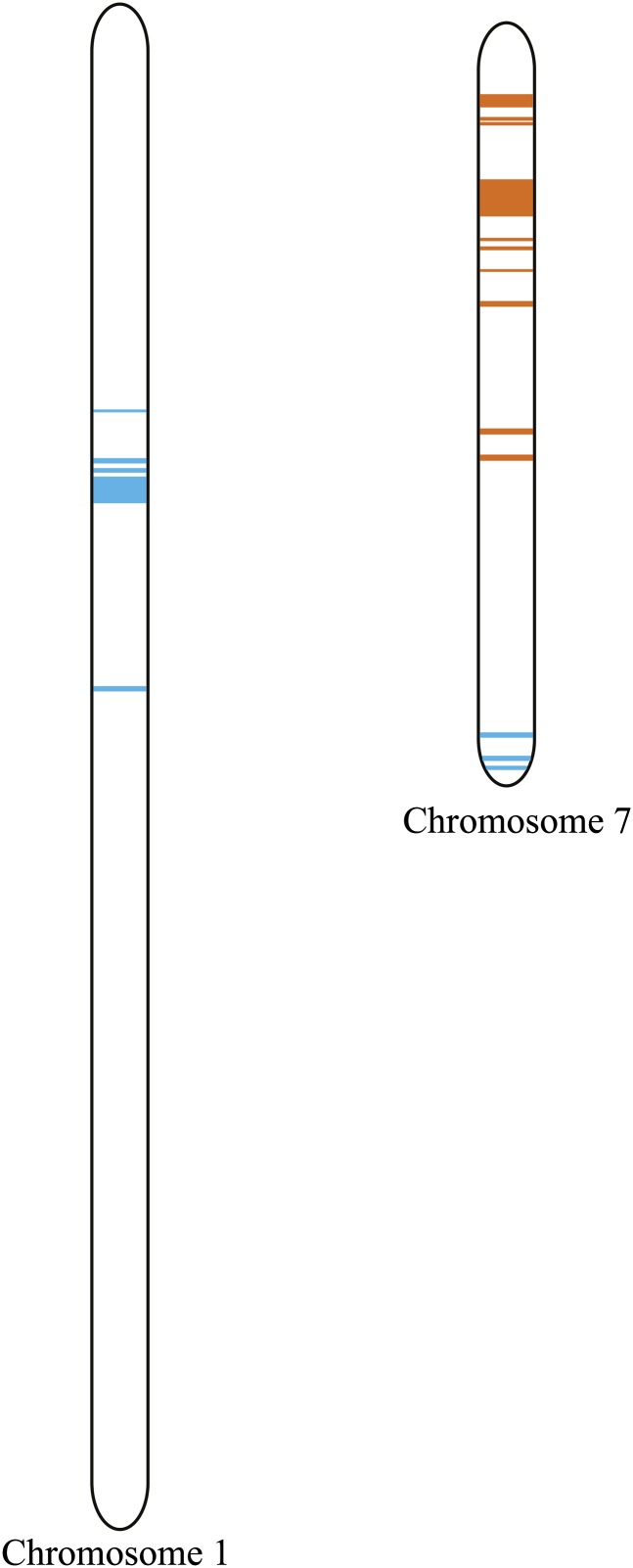
Representation of chromosome 1 and chromosome 7. Blue lines represent Mo17 genotypes, and orange lines indicated B73 genotypes shared among the nine intermated B73 x Mo17 rescuing recombinant inbred line that displayed rescued phenotypes. The largest Mo17 region of chromosome 1 is between 97.5 cM and 99.7 cM. The largest B73 region on chromosome 7 is between 65.7cM and 69cM.

In 2010, a subset of the IBM RIL x *Lgn-R* F1 crosses (IBM18, IBM30, IBM69, and IBM72), which had previously exhibited suppression of the mutant phenotype, was grown at the Purdue Agronomy Center for Research and Education (Table 3 and Figure S1). This subset of IBM RILs rescued much of the *Lgn-R* phenotype, displaying leaf length and width measurements approaching wild-type siblings (Table 3 and Figure S1). In 2011, the same subset of IBM RILs was grown as IBM RIL x *Lgn-R/+* F1 and (IBM RIL x *Lgn-R*) x B73 F1 (a B73 recurrent parent BC1 of the IBM RIL carrying *Lgn-R*) plants were grown at the Gill Tract farm in Albany, CA. Phenotypic rescue was again observed. Thus, over 3 years and in all locations, these RIL were able to rescue the *Lgn-R* mutant phenotype (data not shown). On close examination of the genotypes among these “rescuing” IBM RILs (Table 3 and Figure S1) as well as those RILs identified in Table 1 (IBM4, IBM25, IBM47, IBM55, and IBM65), a region on chromosome 1 between 97.5 cM and 99.7 cM was invariantly Mo17 in these RILs (Figure 4 and Table S2). This region is a subset of the previously identified *sol* interval, which mapped to 94 and 133.5cM. In addition, a region of chromosome 7 between 65.7 cM and 69 cM was invariantly B73 in the rescuing RILs (Figure 4 and Table S2).

**Table 3:** Mean values of leaf morphology traits for “rescuing IBM” RIL x *Lgn-R* F1 deemed “rescued lines” as well as parent lines measured in West Lafayette, IN (2010)

*Lgn-R* F1 Genotype*^a^*	Length*^b^*	Width	Leaf Area	LbW
RILx*Lgn-R*/+ (IBM18)	43.47	4.19	192.45	12.07
RILx*Lgn-R*/+ (IBM30)	41.90	3.86	198.00	12.43
RILx*Lgn-R*/+ (IBM69)	39.53	3.91	168.87	11.67
RILx*Lgn-R*/+ (IBM72)	42.63	3.59	166.65	14.18
B73 (wild type)	77.0	8.92	687.50	8.65
Mo17 (wild type)	65.83	8.33	550.42	7.95

RIL, recombinant inbred line.

a RIL x *Lgn-R/+* F1 genotypes were identified as rescued phenotypes in the 2010 West Lafayette, IN, growing season.

b Length, width, leaf area, and length by width (LbW) were phenotypic measurements made on the 6th leaf from the tassel. Leaf area (length × width) and LbW (length by width) measurements were calculated using length and width measurements.

**Figure 5:**
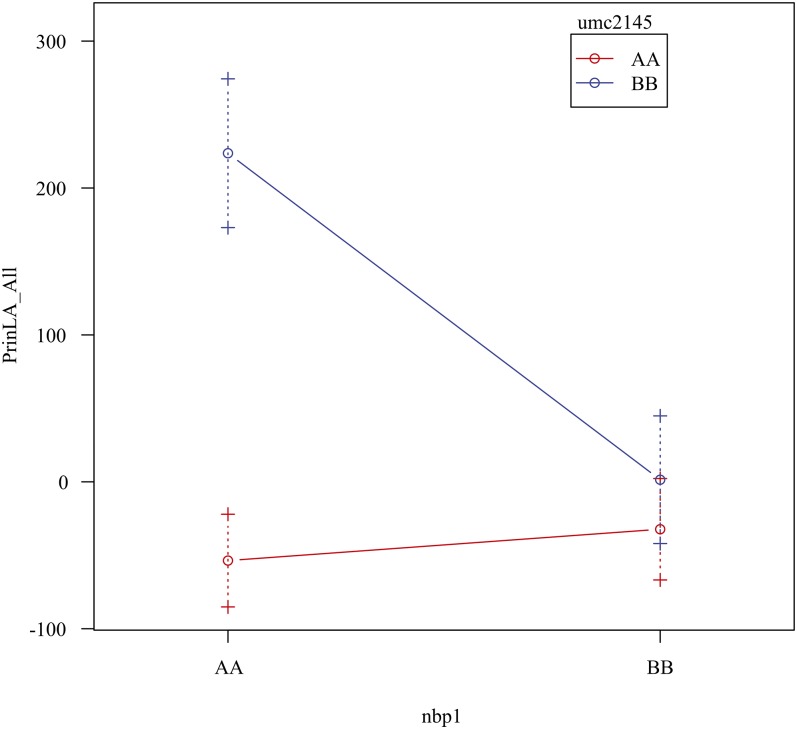
Effect plots (R/qtl; Broman *et al.* 2003) for markers umc2145 (*sol*, chromosome 1) and nbp1 (*lcf*, chromosome 7) for the trait: principal component analysis value of leaf area, all locations. AA indicates the B73 allele and BB indicates the Mo17 allele for marker nbp1. For marker umc2145, the red line represents B73 and the blue line represent Mo17.

We tested for the effect of the region on chromosome 7 on suppression of *Lgn-R* expression while controlling for the effect of segregation at *sol*. Phenotypic data from the full 63 RIL mapping population (Figure 4 and Table S2) were analyzed in pairwise marker regression tests using the marker umc2145 as a proxy for *sol* and each marker in the chromosome 7 segment (Figure 4 and Table S2). Each regression included the umc2145 genotype, the genotype of a marker from chromosome 7, and the pairwise interaction between these two markers. The inclusion of a marker from the chromosome 7 region increased the fit of the model to the data and returned significant *sol* x *lcf* interaction terms indicating an epistatic relationship between the *sol* and *lcf* variants. Thus, this region contains a second QTL interacting with *sol*, which we name *lucifer* (*lcf*). Of all the markers tested within the invariantly B73 region of the rescuing RILs, the marker npb1 had the strongest interaction with *sol*. Figure 5 shows the effect of genotype at *sol* (marker umc2145) and *lcf* (marker nbp1) on the first principal component of leaf measurements. The *sol^Mo17^* allele partially suppresses the effect of *Lgn-R* (Figure 5) and occurs as an invariant region within the rescuing RIL (Figure 4 and Table S2). Consistent with the occurrence of a B73 segment from chromosome 7 in the rescuing RIL (Figure 4), the *lcf^B73^* allele enhances rescue of *Lgn-R* by *sol^Mo17^* (Figure 5). For all phenotypic traits examined, except for stand count data, *lcf* (marker nbp1) modifies *sol* (marker umc2145) such that when *sol*^Mo17^ and *lcf^B73^* are found together, the *Lgn-R* phenotype is substantially suppressed (Figure 5 and Figure S2).

### Two-dimensional genome-wide scan for genetic interactions

The detection of a single pairwise interaction and positive transgressive epistasis demonstrated that we missed QTL due to epistasis in our single-dimension QTL scan. The *scantwo* function (R/qtl, method marker regression) was used to examine all pairwise combinations of markers by regression (Broman *et al.* 2003). Experiment-wide permutation thresholds (1000 permutations, threshold of alpha < 0.05) were calculated from the *scantwo* results for each phenotype. To simplify the output, summaries display only the best QTL-pair for each chromosome pair. Putative interactions were detected two ways. First the data were summarized to display differences between the full model (two QTL model with interactions) and reduced models (*e.g.*, a two QTL model with no interaction; Broman *et al.* 2006). The second method maximized the interaction LOD for a pair of positions on each chromosome, thus returning the pair with the greatest synergistic effect (Broman *et al.* 2006). LODs were calculated for each summary type for each trait. Significant interactions are summarized in Table 4. Almost all interactions above their respective experiment-wide permutation thresholds included the *sol* region on chromosome 1. This finding is not surprising, given the dramatic impact of *sol* and the observation that it was the only main effect QTL identified, thereby returning greater LOD scores for models that include this QTL. Genome-wide multiple testing correction via permutation was used and significant interaction was detected between chromosome 1 at the *sol* QTL and chromosome 7 at *lcf* (Table 4), confirming our observation within the suppressing RIL. *lcf* was detected using the PCA value for length and width using either CA or the PCA from all locations (IN and CA). Additional two-QTL models were significant at unlinked locations on chromosome 1 (Table 4) affecting PCA value for length and width (IN only) and PCA value for leaf area (CA and IN). Lastly, a two-QTL model identified loci on chromosome 2 and chromosome 5 affecting the stand counts for location IN_1 (Table 4). A paralog of *lgn*, *sister of liguleless narrow* (*sln*; GRMZM2G009506), is also located on chromosome 5 but at an unlinked position at the opposite end of the chromosome, excluding the possibility that *sln* encodes this QTL.

**Table 4:** A summary of the maximum interaction LOD scores above an experiment-wide permutation threshold for a two-QTL model (*scantwo*, R/qtl)

	Interaction*^a^*	pos1*^b^*	pos2*^c^*	lod.full*^d^*	lod.fv1*^e^*	lod.int*^f^*
Count data, IN1 location	c2:c5	63.8	83.2	9.86*	7.167	7.46*
PCA value for length and width, IN only	c1:c1	119	134	10.82*	5.11	5.95*
PCA value for length and width, GT only	c1:c4	122	80.9	8.38*	4.87	6.11*
	c1:c7	122	81.4	8.38*	4.87	6.11*
PCA value for length and width, all locations	c1:c3	93.9	189.3	10.6*	5.633	6.29*
	c1:c7	93.4	70.3	11.1*	6.138	8.23*
PCA value for leaf area, all locations	c1:c1	63.3	110	10.3*	6.53	6.53*

GT, xxx; LOD, xxx; QTL, quantitative trait loci; PCA, principal component analysis.

a Shows the two chromosomes with positions that interact in a two-QTL model (*scantwo*, R/qtl).

b Indicates the genome position on the first chromosome listed in the “interaction” column.

c Indicates the genome position on the second chromosome listed in the “interaction” column.

d Lod.full represents values from the full model with QTL (mu+pos1+pos2+(1x2)+error).

e Lod.fv1 represents a comparison of the full model with QTL on chromosome *j* and *k* (assume only 1 QTL on each chromosome).

f Lod.int compares the full model with QTL on chromosome *j* and *k* and indicated interaction between the QTL.

* Denotes statistically significant at the experiment-wide permutation threshold at α = 0.05. One thousand permutations were conducted for each phenotype using *scantwo* (two-QTL model, R/qtl).

### Confirmation of *sol* effects on leaf development in segregating material

In the 2011 field season at Gill Tract Farms (Albany, CA), an evaluation of *sol* and *lcf* effects on leaf and plant morphology was performed in segregating families. Four IBM lines that provided rescue of *Lgn-R* in the aforementioned experiments (IBM18, IBM30, IBM69, and IBM72; Table 3) were backcrossed to B73. Leaf measurements as well as plant height were recorded in the IBM RIL x *Lgn-R/+* BC1 F1s. Individuals were classified as either severe *Lgn-R* mutants, mild expressing mutants, or nonmutants. The genotype of marker umc2145 was used as a proxy for *sol*. The markers bnlg1792 and npb1 are both located at 66 cM on chromosome 7 and were used to confirm the *lcf* genotype, which was not segregating in these families because of the homozygosity for the *lcf^B73^* allele in the RIL and B73 being the recurrent parent. A genotype of either B73, Mo17, or B73/Mo17 heterozygote was noted for each locus in each individual (File S1). The effects of the IBM RIL genetic background, *sol* genotype, *Lgn-R* mutation, and the interaction of *Lgn-R* and *sol* were estimated using a Standard Least Squares model for each of the three phenotypes: leaf length, leaf width, and plant height (Table 5). The genetic background effect is defined by the filial generation and genetic background (*e.g.*, B73, Mo17, or the 63 RILs) separate from the genotype at *sol*. The *sol* effect estimates the significance of the genotype at umc2145. Finally, the effect the genotype at *lgn* was also included, as these familes are segregating 1:1 for *Lgn-R*/+ and wild-type *lgn* homozygotes. All three primary parameter estimates as well as the interaction of *sol* with *Lgn-R* were statistically significant (Table 5). The interaction between the *sol* genotype and mutant phenotype indicate that *sol* impacts phenotype within the IBM x *Lgn-R*/+ individuals to a greater degree than in *lgn* wild-type siblings.

**Table 5:** Parameter estimates for B73, IBM x *Lgn-R* F1 and IBM x *Lgn-R* BC1 individuals using the Standard Least Squares model

Model Parameters	Leaf Width*^a^*				Leaf Length*^a^*			Plant Height*^a^*		
	DF*^b^*	SS	F Ratio	*P* Value	SS	F Ratio	*P* Value	SS	F Ratio	*P* Value
Background*^c^*	7	217.76	19.18	<0.001***	5624.89	12.62	<0.001***	169333.45	27.2039	<0.001***
*sol**^d^*	1	39.26	24.21	<0.001***	553.381	8.69	0.0036**	14577.19	16.39	<0.001***
Mutant*^e^*	1	747.73	461.06	<0.001***	16772.33	263.32	<0.001***	92337.60	103.84	<0.001***
Mutant x *sol*	1	54.17	33.40	<0.001***	917.84	14.41	0.0002***	5003.19	5.63	0.0188**
Whole model	10	1498.64	92.41	<0.001***	29971.55	47.05	<0.001***	480587.93	54.05	<0.001***
Error	172	278.94			10955.71			153836.84		
Total	182	1777.58			40927.26			634424.78		

DF, degrees of freedom; REML, restricted maximum likelihood; SS, xxx.

a Leaf length and width, as well as and plant height measurements, were made in 2011 (Gill Tract Farm; Albany, CA) for IBM x *Lgn-R*/+ individuals.

b DF calculated from a REML model. DFs are the same for each phenotype and are thus listed once.

c Different backgrounds were defined in 2011 data: B73, B73/Mo17, and IBM x *Lgn-R*/+ F1 individuals.

d sol is defined as the genotype of IBM x *Lgn-R*/+ individuals using marker umc2145. Genotypes are defined as B73, Mo17 or heterozygous B73/Mo17.

e BC1 IBM x *Lgn-R*/+ individuals were observed to be mutant (*Lgn-R*-like) or wild type (IBM sibling-like).

** Denotes statistical significance at α = 0.01.

*** Denotes statistical significance at α = 0.001.

### Genotype x environment interaction

Genotype by environment (GxE) interactions were estimated in the 63 IBM RIL mapping crosses using a Standard Least Squares model. We determined the effects of *sol* and *lcf* and their interaction (using markers umc2145 and nbp1, respectively) while also considering the growth environments as factors. Across all environments (CA and IN), we determined GxE and (GxG) x E interaction (Table 6). The interactions between *sol* and *lcf* (*P* < 0.0001) as well as *sol* x *lcf* x environment (*P* < 0.0003) were both statistically significant (Table 6). If we examine the leaf area phenotype within each location, *sol* was significant in both environments (*P* = 0.0223, CA and IN both needed) but the interaction between *sol* and *lcf* was only detected in IN (*P* < 0.001; Table 7). It appears that some aspect of the environment or cultural practice that differs between the IN and CA field sites influences the expression of *Lgn-R* phenotypes and with it *lcf* and the interaction effect of *lcf* and *sol*.

**Table 6:** Examination of main effects of *sol*, *lcf*, and location as well as genotype x environment (GxE) interactions calculated for leaf area across locations (IN and CA) using the 2009 dataset

Model Parameters	DF*^a^*	Leaf Area, CA and IN		
		SS	F Ratio	*P* Value
*lcf* (nbp1)	1	34059.67	19.6343	<0.0001***
*sol* (umc2145)	1	127130.14	73.2865	<0.0001***
*lcf* x^1^*sol*	1	60631.45	34.9521	<0.0001***
Location	2	260913.90	75.2043	<0.0001***
*lcf* x location*^b^*	2	17149.37	4.9430	0.0087**
*sol* x location	2	66084.31	19.0478	<0.0001***
*lcf* x *sol* x location	2	30825.07	8.8848	0.0003***
Whole model	11	439921.61	23.05	<0.0001***
Error	117	202960.01		
Total	128	642881.63		

a DF is the degrees of freedom for each parameter in the model. The full model and error DFs are included.

b x indicates interaction between two or more parameters within the model. Genotype of *sol* and *lcf* were used for GxE; IN and CA define the location parameter. Main effects and interactions were calculated using a Standard Least Squares model (JMP, version 8.0.1, SAS Institute Inc., 2009).

** Denotes statistical significance at α = 0.01.

*** Denotes statistical significance at α = 0.001.

**Table 7:** Examination of 2009 data set for main effects of *sol*, *lcf*, and location as well as genotype x environment (GxE) interactions calculated for leaf area, treating locations separately

Model Parameters		Leaf Area, CA Only				Leaf Area, IN1 Only				Leaf Area, IN2 Only		
	DF*^a^*	SS	F Ratio	*P* Value	DF	SS	F Ratio	*P* Value	DF	SS	F Ratio	*P* Value
*lcf* (nbp1)	1	0.506	0.421	0.520	1	22791.24	9.05	0.0048***	1	27504.46	9.314	0.0041**
*sol* (umc2145)	1	6.76	5.620	0.022*	1	94779.07	37.62	<0.0001***	1	94848.29	32.12	<0.0001***
*lcf* x *sol**^b^*	1	0.103	0.086	0.771	1	40394.14	16.03	0.0003***	1	49433.73	16.74	0.0002***
Whole model	3	7.24	2.01	0.127	3	114191.17	15.11	<0.0001***	3	120441.38	13.60	<0.0001***
Error	43	51.703			36	90692.98			38	112215.33		
Total	46				39	204884.15			41	232656.71		

a DF is the degrees of freedom for each parameter in the model. The full model and error DFs are included.

b x indicates interaction between two or more parameters within the model. Genotype of *sol* and *lcf* were used for genotype x environment (GxE); IN and CA define the location parameter. Main effects and interactions were calculated using a Standard Least Squares model (JMP, version 8.0.1, SAS Institute Inc., 2009).

* Denotes statistical significance at α = 0.05.

** Denotes statistical significance at α = 0.01.

*** Denotes statistical significance at α = 0.001.

### Leaf morphology QTL comparison

Tian and Co-Workers (2011) previously carried out a genome-wide QTL mapping experiment for leaf morphology using the nested-association panel, which includes a Mo17-B73 contrast. We compared our *sol* QTL region (94 cM to 133.5 cM on the IBM ISU v4 map; Table S1) with the Tian *et al.* (2011) genome-wide association study single-nucleotide polymorphism (SNP)/Indel collection. For clarity, both the Tian *et al.* (2011) SNP positions within the *sol* QTL and IBM ISU v4 map positions defining the *sol* locus are provided as maize assembly AGPv1 corrdinates in Table 8 (B73 v1 map; http://www.maizegdb.org; Schnable *et al.* 2009). All SNPs with significant associations to leaf traits in Tian *et al.* (2011) within the broad *sol* interval on chromosome 1, 54,036,789−183,652,422 bp (94−133.5 cM; Table S1), are listed in Table 8. The narrowed invariant Mo17 region within *sol* identified by the rescuing RILs (Table 3, Figure 4, and Table S2) from 97.5 cM and 99.7 cM was converted to 62,995,272 bp to 66,027,760 bp on chromosome 1 in the AGPv1 build. Not all SNPs could be mapped to AGPv2; those SNPs that were unambiguously mapped to locations in the AGPv2 maize assembly (http://www.maizesequence.org) also are presented in Table 8. In total, four joint-linkage analysis QTL, 21 significant GWA SNPs, and 1 indel fell within the Bayesian credible interval for *sol* for leaf angle, leaf length, or leaf width. The narrowed QTL region did not include any QTL or SNP/indels from the work of Tian and *et al.* (2011). Also, no trait-associated SNPs (Yu *et al.* 2012) for leaf morphology traits overlapped with *sol*. Likewise, of the leaf morphology QTL identified in the IBM population by Mickelson *et al.* (2002), none were linked to *sol*. Thus, by using natural variation to search for mutant suppressors and genetic interaction, we have detected the gene, *sol*, for leaf morphology. In addition to detecting *sol*, we have also detected an interacting locus, *lcf*, opening up the possiblity of constructing a genetic pathway including the products of *lgn*, *sol*, and *lcf* all heretofore unknown determinants of leaf growth and architecture in maize.

**Table 8:** QTL identified for leaf architecture located within the Bayesian credible interval for the *lgn* QTL

	QTL*^a^*	SNP/Indel	AGPv1 Position, bp*^b^*^,^*^c^*	B73v2 Position*^d^*	Chr*^b^*	Pos, cM*^b^*	Allele*^b^*	*P* Value*^b^*	Effect*^b^*	Polymorphic between B73 and Mo17*^e^*
Leaf angle*^f^*	m82	PZE01114045567	114045567	115217353	1	89.088	A/G	1.30E−11	0.59	A/G (yes)
	m82	PZE01118507354	118507354	na*^g^*	1	89.279	G/−	4.00E−13	0.70	G/− (?)
	m82	PZE01148006384	148006384	na	1	89.595	T/C	2.10E−11	0.90	T/C (yes)
	m82	PZE01173899899	173899899	174030908	1	94.380	G/A	3.00E−17	0.69	G/A (yes)
Leaf length	m52	PZE0151533532	51533532	51403200	1	66.023	G/T	5.40E−13	5.06	G/T (yes)
	m52	PZE0151575729	51575729	na	1	66.074	C/T	1.90E−12	5.65	C/C (no)
	m52	PZE0152025282	52025282	na	1	66.624	G/C	4.50E−11	5.12	G/− (?)
	m69	PZE0182570732	82570732	83787495	1	82.402	T/G	1.40E−19	5.78	T/G (yes)
	m69	PZE0182570902	82570902	na	1	82.402	−/ACGT	3.40E−15	5.53	−/− (?)
	m69	PZE0192531865	92531865	na	1	85.471	T/C	1.40E−24	6.69	T/T (no)
	na*^h^*	PZE01101787357	101787357	na	1	87.956	C/T	5.00E−08	6.25	C/C (no)
	na	PZE01102256886	102256886	na	1	88.027	−/A	9.00E−08	7.06	−/− (?)
	na	PZE01136166765	136166765	na	1	89.474	T/C	1.10E−06	−7.58	T/C (yes)
Leaf width	m56	PZE0152655271	52655271	na	1	67.395	G/C	1.90E−14	1.29	G/N (?_
	m56	PZE0153509826	53509826	53422803	1	68.440	G/A	6.60E−08	0.59	G/G (v1) OR G/A (v2)
	m56	PZE0159424192	59424192	59358936	1	71.602	C/G	4.90E−09	0.95	C/C (no)
	na	PZE01109523099	109523099	na	1	88.813	T/C	9.10E−08	0.96	T/N (?)
	na	PZE01139538038	139538038	na	1	89.509	A/C	6.10E−12	1.05	N/N (?)
	na	PZE01146105232	146105232	147197048	1	89.575	C/A	2.20E−08	−1.25	C/A (yes)
	na	PZE01148471044	148471044	148404129	1	89.599	T/C	8.10E−10	−1.07	T/C (yes)
	na	PZE01150965828	150965828	150898913	1	90.012	G/C	3.70E−13	1.72	G/G (no)
	na	PZE01153739401	153739401	153696642	1	90.393	A/G	8.10E−17	1.95	A/A (no)

QTL, quantitative trait loci; SNP, single-nucleotide polymorphism.

a QTL in which the SNP or indel falls within the supportive interval based on the cM position, as defined by Tian *et al.* (2011).

b The AGPv1 position, allele, *P* value, and effect are all taken from Tian *et al.* (2011).

c Each SNP or indel listed falls within the Bayesian credible interval (51409853 bp and 173228669 bp) for chromosome 1 for AGPv1 map.

d The SNP location is also listed from the B73 v2 position from http://www.maizegdb.org.

e B73 and Mo17 genotypes at indicated SNP position was determined from the Hap map v1 or v2 (http://www.panzea.org). SNPs polymorphic between B73 and Mo17 are indicated as “yes.”

f QTL were previously identified by Tian *et al.* (2011) and are partitioned in the three different phenotypes: leaf angle, length, and width.

g Indicates that SNPs have yet to be mapped to B73v2 map.

h Indicates the SNP or indel listed falls outside the QTL supporting interval position (cm).

## Discussion

We have used natural variation affecting the penetrance of the leaf morphology traits conferred by the semidominant *Lgn-R* mutant to identify a locus on the short arm of chromosome 1, *sol*. In addition to the *sol* locus, two-dimensional QTL scans identified a second locus, *lcf*, on chromosome 7 that interacts with *sol* to determine the overall degree of phenotype rescue in *Lgn-R* mutants. *Lgn-R* was shown previously to affect ligule and auricle development (Moon *et al.* 2013) but can also cause termination of the development of the maize plant before ear and tassel production. In the 2009 field season, IBM RIL x *Lgn-R/+* F1 individuals grown in two different locations, IN and CA, were used to detect and localize *sol* and *lcf* by regression-based QTL analysis methods. The *sol* QTL is a novel gene as recent maize QTL mapping experiments (Mickelson *et al.* 2002; Tian *et al.* 2011; Yu *et al.* 2012) identified QTL affecting leaf morphology not contained within the narrow interval for *sol* defined by the IBM RILs that rescued the *Lgn-R* phenotype to near wild-type leaf measures (Table 3, Figure 4, Figure S1, and Table S2).

Four IBM RIL x *Lgn-R*/+ F1s displayed a near-rescued phenotype in 3 years at four locations (IBM18, IBM30, IBM69, and IBM72; Table 3 and Figure S1). These RILs, as well as five others with near wild-type leaf measurements from the 2009 RIL mapping experiments (IBM4, IBM25, IBM47, IBM55, and IBM65; Table 1), are invariantly Mo17 at the *sol* locus. We referred to these at the “rescuing RILs.” Likewise, these nine IBM RIL had B73 alleles at the location of *lcf* (Figure 4 and Table S2). These near wild-type phenotypes were recorded at four locations in 3 years both as IBM RIL x *Lgn-R/+* crosses and as B73 x (RIL x *Lgn-R*/+) BC1 F1 plants for IBM18, IBM30, IBM69, and IBM72, indicating the additivity or dominance of *sol^Mo17^*.

The marker effect plots for umc2145 and npb1 linked to *sol* and *lcf* demonstrate transgressive positive-epistasis such that the optimum combination of *sol^Mo17^/lcf^B73^* is significantly rescued and all other combinations are indistiguishable (Figure 5 and Figure S3). The fact that these interactions occur in IBM RIL x *Lgn-R/+* F1 material and that *Lgn-R* was isolated in a B73 background further support the additive or dominant nature of *sol^Mo17^* but do not clarify whether *lcf^B73^* is additive or recessive. *Lgn-R* x Mo17 BC1 and BC2 generations exhibited partially suppressed *Lgn-R* phenotypes (data not shown). This finding is consistent with *lcf* enhancing the *sol^Mo17^* suppression of *Lgn-R* but not being required for *sol^Mo17^* expression.

The interaction between *sol* and *lcf* was demonstrated in the marker effect tests as well as the genotypes of a set of fully suppressed RIL x *Lgn-R/+* crosses. All phenotypes except stand counts in CA (count_GT) are closer to wild type when the *sol* allele (umc2145) is Mo17 (blue line; Figure 5 and Figure S2). However, *sol^B73^* IBM RIL x *Lgn-R/+* F1s have narrower leaves much like the original B73 *Lgn-R* isolate described by Moon *et al.* (2013) (Figure 1). Similarly, the *lcf* alleles impact all phenotypes coordinately and the *lcf^B73^* allele always promoted wild-type phenotype expression (Figure 5).

Because the loss-of-function allele at *lgn* had no visible phenotype in Albany, CA, the *Lgn-R* allele is predicted to be an antimorph (Moon *et al.* 2013). Thus, *trans*-regulatory polymorphisms increasing transcription from the *lgn* locus would be expected to enhance the *Lgn-R* phenotype. *Lgn-R/+* plants exhibit increased accumulation of the mRNA encoded by the paralog, *sln*, in shoot apices whereas loss-of-function alleles did not alter *sln* mRNA accumulation (Moon *et al.* 2013). The expression levels of *lgn* and *sln* in RNAseq data from B73 and Mo17 were made available by the Pat Schnable lab at Iowa State University, ahead of publication, via James Schnable’s qTeller tool (http://qteller.com/). Consistent with the penetrance of the *Lgn-R* phentypes in B73 and Mo17, *lgn* mRNA is accumulated at a greater level in the apices of B73 as compared with Mo17 (Figure S3). The opposite expression difference was observed for *sln*, which exhibited a greater accumulation of mRNA in RNA isolated from Mo17 shoots than was observed for B73 (Figure S4).

Integration of QTL, genetic interaction, and mRNA expression data provide a potential molecular mechanism for the modulation of leaf morphology and shoot development by *sol* and *lcf*. The *sol* QTL on chromosome one could be explained by an allele at a regulatory gene in Mo17 that suppresses the expression of *lgn*, resulting in suppression of the *lgn* phenotype in the presence of the dominant mutant allele. The presence of multiple splice forms of *lgn* (http://www.maizesequence.org) make this a possible candidate mechanism for the *sol* QTL, as well. Alternatively, expression difference need not underlie the *sol* or *lcf* QTL. Coding sequence changes to known genes or the presence of coding sequence in the Mo17 chromosome not present in the B73 reference, and therefore unannotated for expression, could be responsible for the suppression of *Lgn-R*. The expression of *sln* is greater in shoots of Mo17 and suppression of *sln* expression by the B73 allele at *lcf* could provide the mechanism for *sol^Mo17^*-dependent mutant phenotype suppression by that QTL. The hypothesis of a *lgn*-affecting regulatory QTL at *sol* is consistent with the requirement of *sol^Mo17^* for the expression of *lcf^B73^* and the previous proposal that *lgn* phenotype manifestation is due to up regulation of *sln* in the shoot apex by the dominant *Lgn-R* allele (Moon *et al.* 2013). Testing of candidates genes for *sol* and *lcf*, and the roles they may play in the expression regulation and alternative splicing of *lgn* and the unusual effect of the 8bp UTR insertion allele *lgn-dAc* (Moon *et al.* 2013), await future experiments.

Neither *lcf* nor the interaction between the *sol* and *lcf* QTL were detected in CA (Table 1 and Figure 3). Multiple leaf morphology measures differed between the growing areas used throughout the 4 years of experimentation. According to the National Oceanic and Atmospheric Administration, the mean maximum temperatures during the summer of 2009 indicate that of the two sites used, IN was 6−13°F warmer (Figure S5) than Gill Tract, CA. The expressivity of the *lgn* phenotype was strongest in West Lafayette, IN, than any of the other locations. The penetrance of the *Lgn-R* allele was so strong that gross morphological and quantitative measures of phenotype expression in IN look similar to the *Lgn-R* homozygotes in other areas. For example, heterozygous *Lgn-R/+* plants exhibit a complete failure of all reproductive structures in the B73 background in West Lafayette, IN. Although any differences in these two locations including soil characteristics and management techniques could also explain the lack of *lcf* detection in this CA location. As compared with the Gill Tract, CA site, stronger expression of *Lgn-R* suppression was observed in the 2010 growing season in IN (West Lafayette), during the 2012 season in Davis, CA, and in Valle de Banderas, Nayarit, MX during the winter of 2012 (data not shown). All three of these sites were substantially warmer than the Gill Tract Farm in Albany, CA. Regardless of the cause, the elucidation of a novel genetic pathway affecting leaf morphology and plant architecture including *Lgn-R*, *lcf*, and *sol*, and multiple epistatic and environmental interactions was identified in this study of natural variation affecting expression of a dominant mutant phenotype.

## Supplementary Material

Supporting Information
