# Supplementary material for: Natural Variation at sympathy for the ligule Controls Penetrance of the Semidominant Liguleless narrow-R Mutation in Zea mays
Source: G3 (Bethesda). 2014 Oct 24;4(12):2297–306. doi: 10.1534/g3.114.014183 (PMC4267926; doi:10.1534/g3.114.014183)
Supplement: Supporting Information [file supp_4_12_2297__index.html]

Natural Variation at sympathy for the ligule Controls Penetrance of the Semidominant Liguleless narrow-R Mutation in Zea mays — Supporting Information 

# Natural Variation at *sympathy for the ligule* Controls Penetrance of the Semidominant *Liguleless narrow-R* Mutation in *Zea mays*

## Supporting Information for Buescher *et al.*, 2014

**Files in this Data Supplement:**

- Supporting Information - Figures S1-S5, Tables S1-S2, and Files S1-S2 (PDF, 1 MB)
- Figure S1 - Leaf length and width measurements for five IBM RIL X *Lgn-R*/+F1 individuals displaying near wild-type B73 phenotypes or rescued (indicated in blue) and five individuals displaying near *Lgn-R* phenotypes or suppressed (indicated in red). (PDF, 134 KB)
- Figure S2 - Effect plots (R/qtl Broman et al 2003) for each trait comparing sol (umc2145) and lcf (nbp1) markers. (PDF, 314 KB)
- Figure S3 - Histogram of RNAseq data describing mRNA accumulation for *Lgn* (GRMZM2G134382) in multiple tissue tyes for both B73 and Mo17. (PDF, 241 KB)
- Figure S4 - RNAseq expression data for *Sln* (GRMZM2G009506) in B73 and Mo17 across multiple tissue types. (PDF, 235 KB)
- Figure S5 - Average high temperature data (http://www.noaa.gov) for four growing locations. (PDF, 130 KB)
- Table S1 - The genetic and physical positions of markers delimiting the *sol* QTL. (PDF, 57 KB)
- Table S2 - Genotypes and marker positions of IBMs of interest examined near *sol* (umc2145) and *lcf* (nblg1247) genotypes based on the IBM ISU map version 4. (PDF, 151 KB)
- File S1 - .R, 5 KB
- File S2 - .csv, 444 KB
