# Supplementary material for: Natural Variation at sympathy for the ligule Controls Penetrance of the Semidominant Liguleless narrow-R Mutation in Zea mays
Source: G3 (Bethesda). 2014 Oct 24;4(12):2297–306. doi: 10.1534/g3.114.014183 (PMC4267926; doi:10.1534/g3.114.014183)
Supplement: Supporting Information [file supp_g3.114.014183_014183SI.pdf]

G3: Genes | Genomes | Genetics

Natural variation at *sympathy for the ligule* controls penetrance of the semidominant *Liguleless narrow-R* mutation in *Zea mays*

Elizabeth M. Buescher[*], Jihyun Moon[§], Anne Runkel[§], Sarah Hake[§], and Brian P. Dilkes[*,1]

[*]Department of Horticulture and Landscape Architecture, Purdue University, 625 Agricultural Mall Drive, West Lafayette, IN 47907, United States

[§]Plant Gene Expression Center, USDA-ARS, University of California, Berkeley, 800 Buchanan St, Albany, CA 94710, United States

[1] Corresponding author: Dilkes, Brian P. (bdilkes@purdue.edu)

Brian P. Dilkes

Purdue University, Department of Horticulture and Landscape Architecture

West Lafayette, IN 47907

Phone: (765)494-9042

bdilkes@purdue.edu

DOI: 10.1534/g3.114.014183

Supplemental Figure 1. Leaf length and width measurements for five IBM RIL X *Lgn-R*/+ F1 individuals displaying near wild-type B73 phenotypes or rescued (indicated in blue) and five individuals displaying near *Lgn-R* phenotypes or suppressed (indicated in red). Measurements were made in triplicate in 3 locations: two independent plantings in West Lafayette, IN (Purdue University Agronomy Center for Research and Education) and Albany, CA (University of California Gill Tract Farm). The individuals indicated (IBM18, IBM30, IBM69 and IBM72) are IBM RIL X *Lgn-R*/+ F1 individuals that are phenotypically near wild-type Mo17 in leaf length and width.

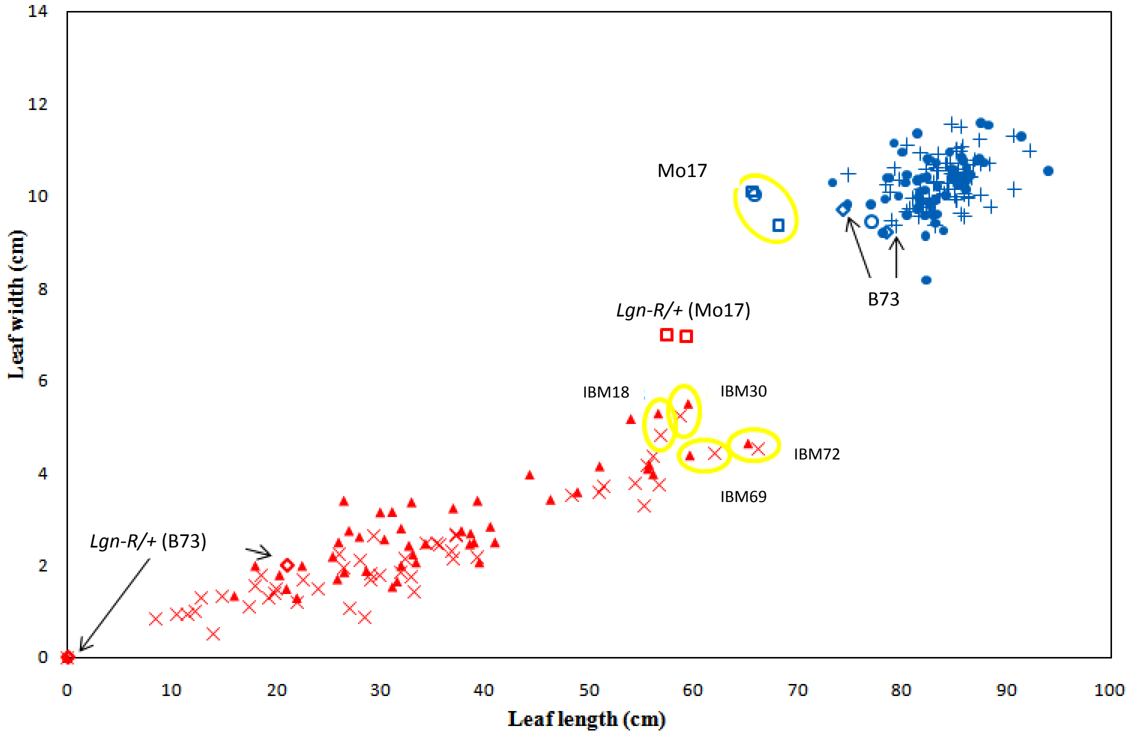

Supplemental Figure 2. Effect plots (R/qtl Broman et al 2003) for each trait comparing sol (umc2145) and lcf (nbp1) markers. The B73 allele (AA) and the Mo17 allele (BB) are indicated on the x-axis for lcf (nbp1). sol (umc2145) is represented by a red line for B73 and a blue line for Mo17.**A-C.** Effect plots for the count data for the **A.** GT location; **B.** IL1 location; **C.** IN 2 location. **D-F** are effect plots for the length and width data: **D.** PCA value for length and width, IN only; **E.** PCA value for the length and width measurements, GT only; **F.** PCA value for the length and width measurements, all locations. **G-H.** effect plots for the leaf area data: **G.** leaf area measurements for GT only; **H.** PCA value for IN.

S2A

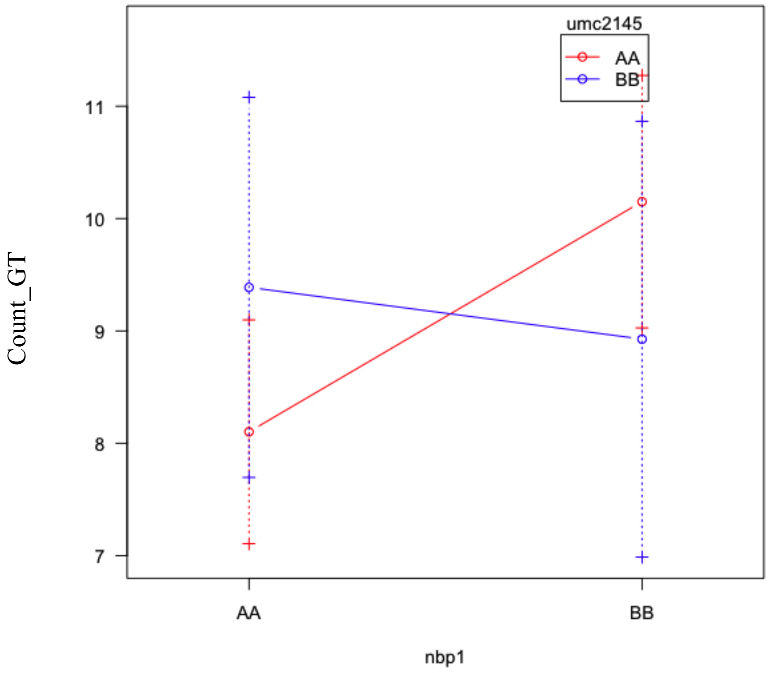

S2B

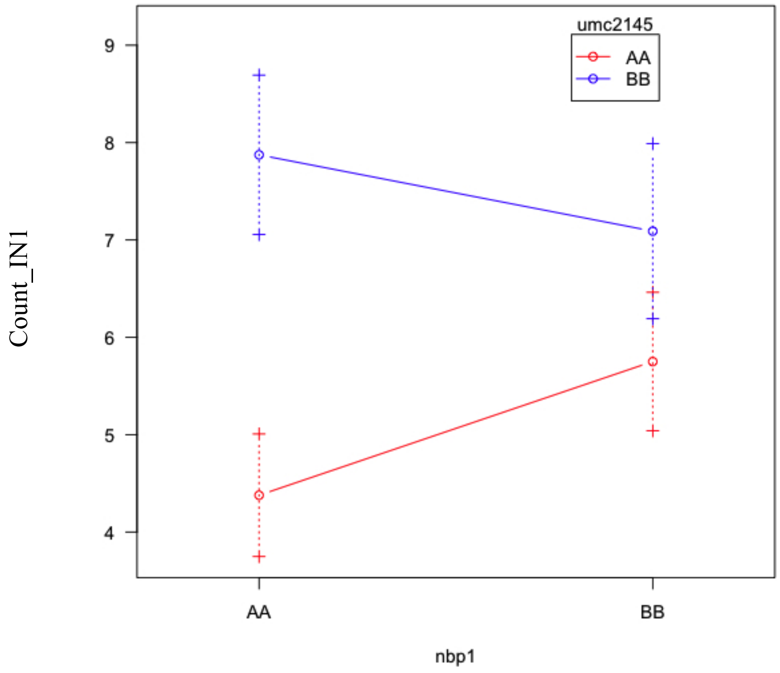

S2C

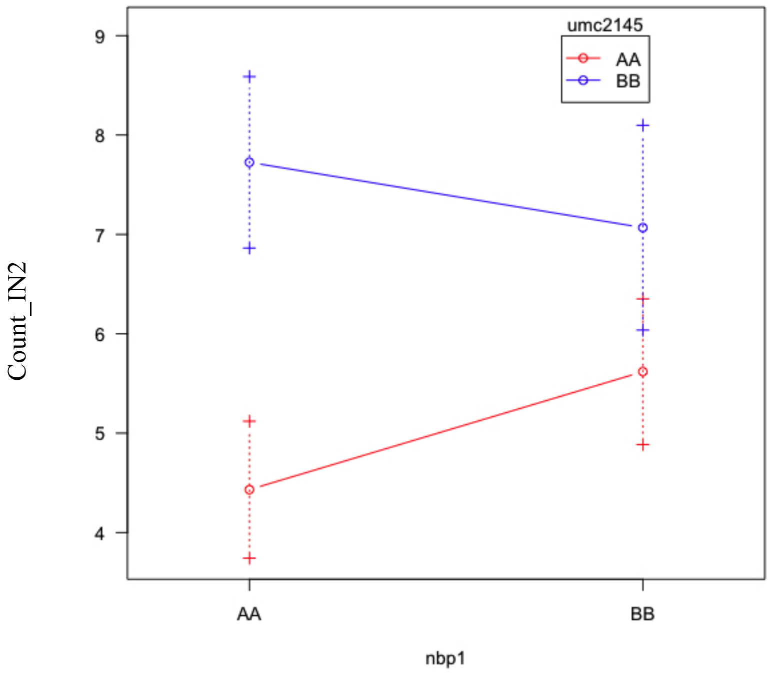

umc2145
AA
BB
9
8
7
6
5
4
Count_IN2
AA
BB
nbp1

S2D

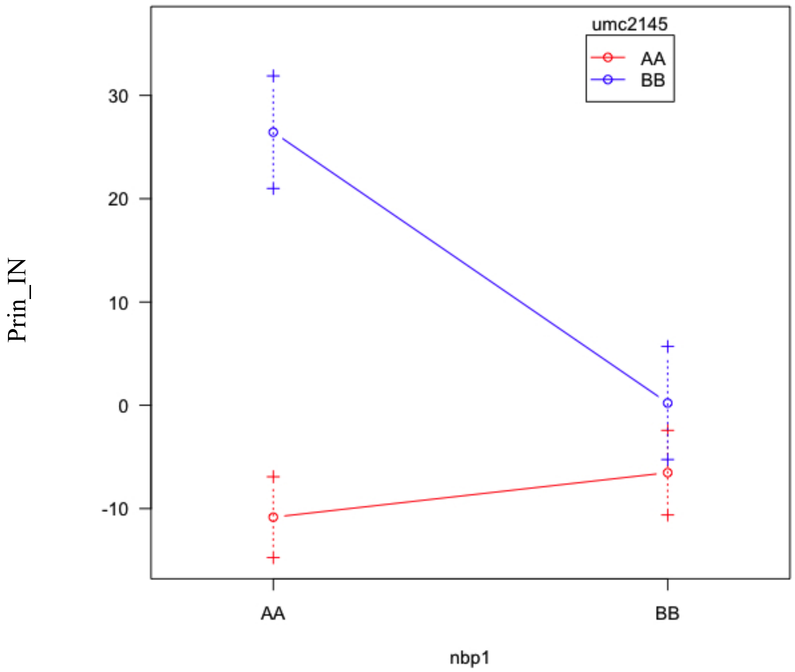

umc2145
AA
BB
30
20
10
0
-10
Prin_IN
AA
BB
nbp1

S2E

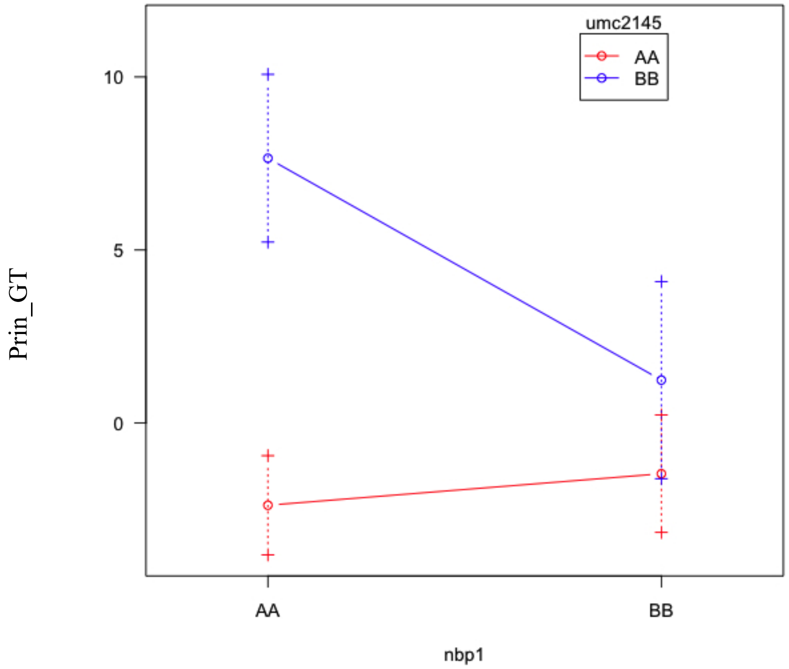
umc2145
AA
BB
10
5
0
Prin_GT
AA
BB
nbp1

S2F

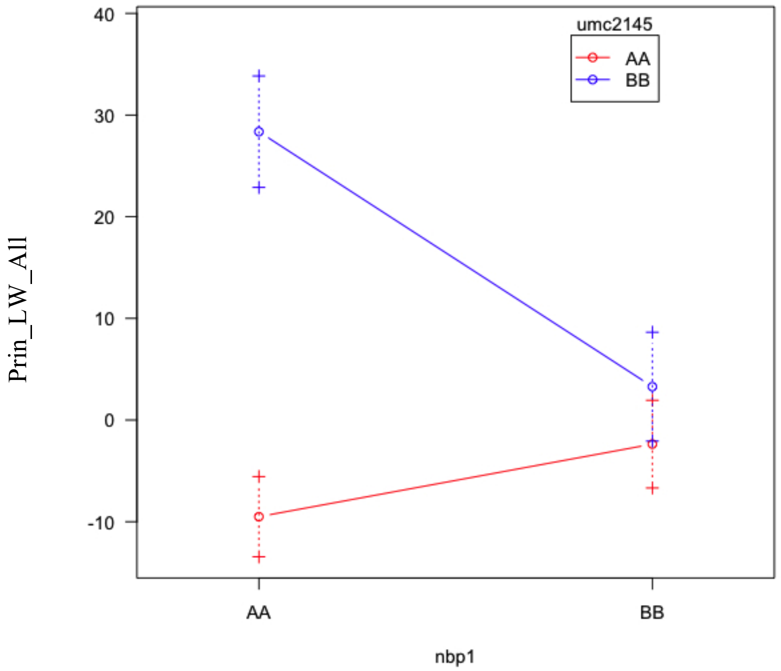
umc2145
AA
BB
40
30
20
10
0
-10
Prin_LW_All
AA
BB
nbp1

S2G

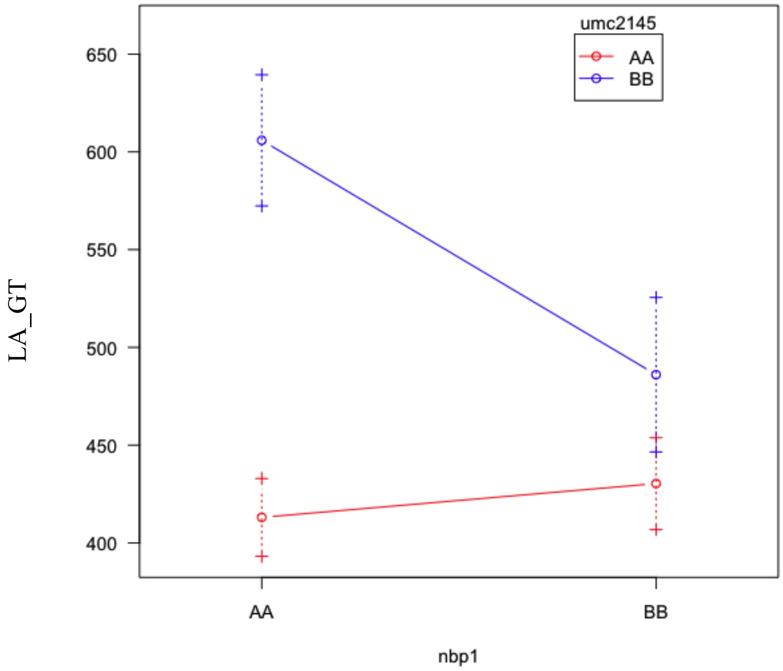
umc2145
AA
BB
650
600
550
500
450
400
LA_GT
AA
BB
nbp1

S2H

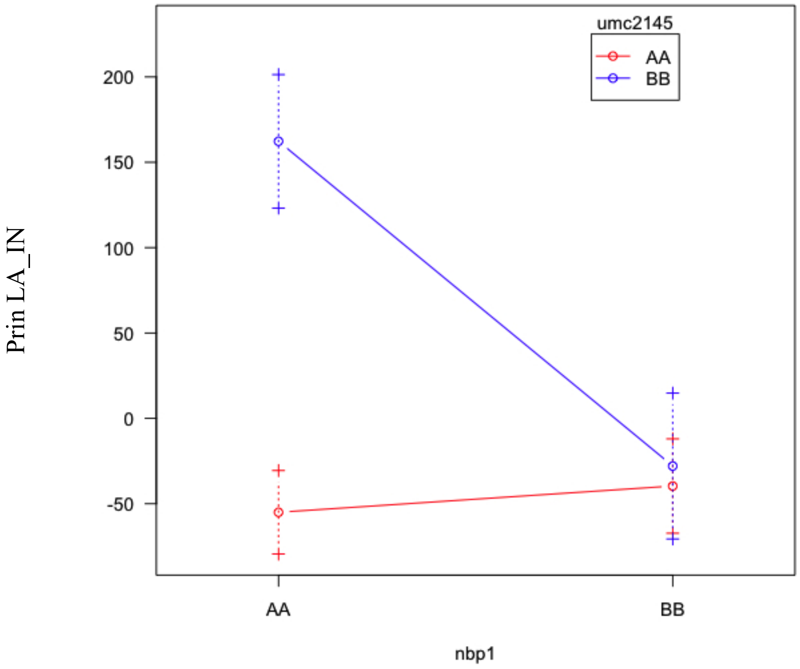
umc2145
AA
BB
200
150
100
50
0
-50
Prin LA_IN_
AA
BB
nbp1

Supplemental Figure 3. Histogram of RNAseq data describing mRNA accumulation for *Lgn* (GRMZM2G134382) in multiple tissue tyes for both B73 and Mo17. RNAseq data was generated using the qTeller tool (http://qteller.com/). Tissue types are as follows: root, shoot, tassel and shoot apex. The HTML link for regenerating this analysis at the qTeller website is:

http://qteller.com/NAM/bar_chart.php?name=GRMZM2G134382&info=B73_root%7CMo17_root%7CMo17_sam_apex%7CB73_sam_apex%7CMo17_tassel%7CB73_tassel%7CMo17_shoot%7CB73_shoot

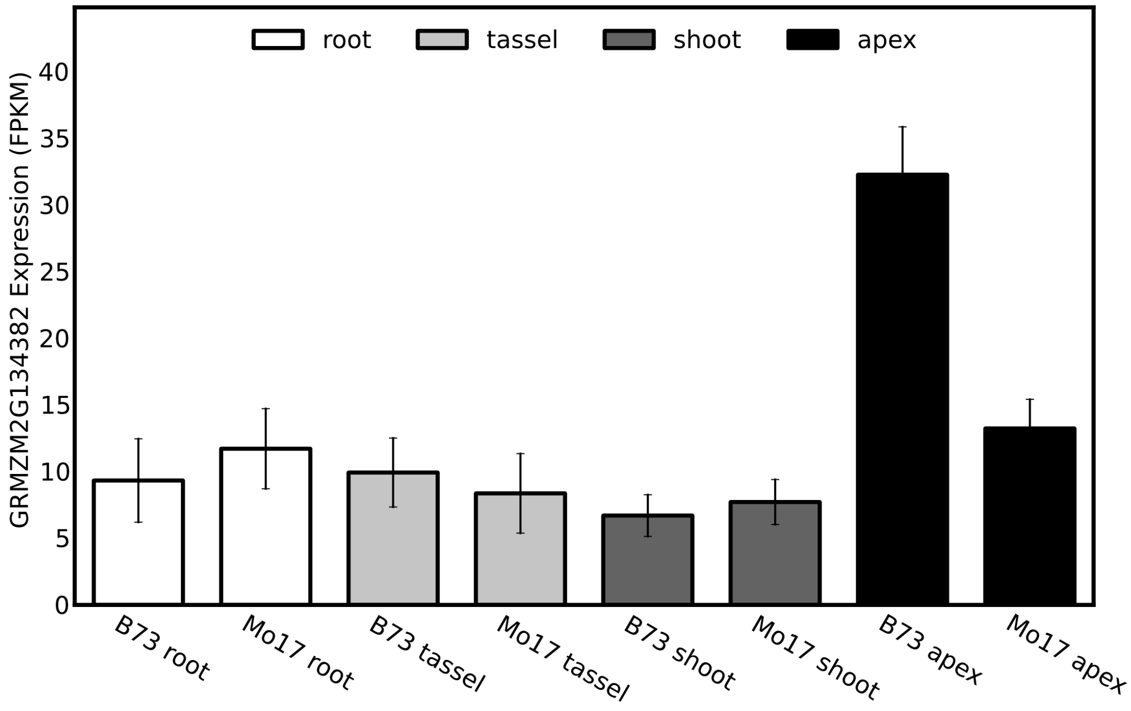

Supplemental Figure 4. RNAseq expression data for *Sln* (GRMZM2G009506) in B73 and Mo17 across multiple tissue types. mRNA data was generated using qTeller (http://qteller.com/). Tissue types are as follows: root, shoot, tassel and shoot apex. The HTML link for regenerating this analysis at the qTeller website is: http://qteller.com/NAM/bar_chart.php?name=GRMZM2G009506&info=B73_root%7CMo17_root%7CMo17_sam_apex%7CB73_sam_apex%7CMo17_tassel%7CB73_tassel%7CMo17_shoot%7CB73_shoot

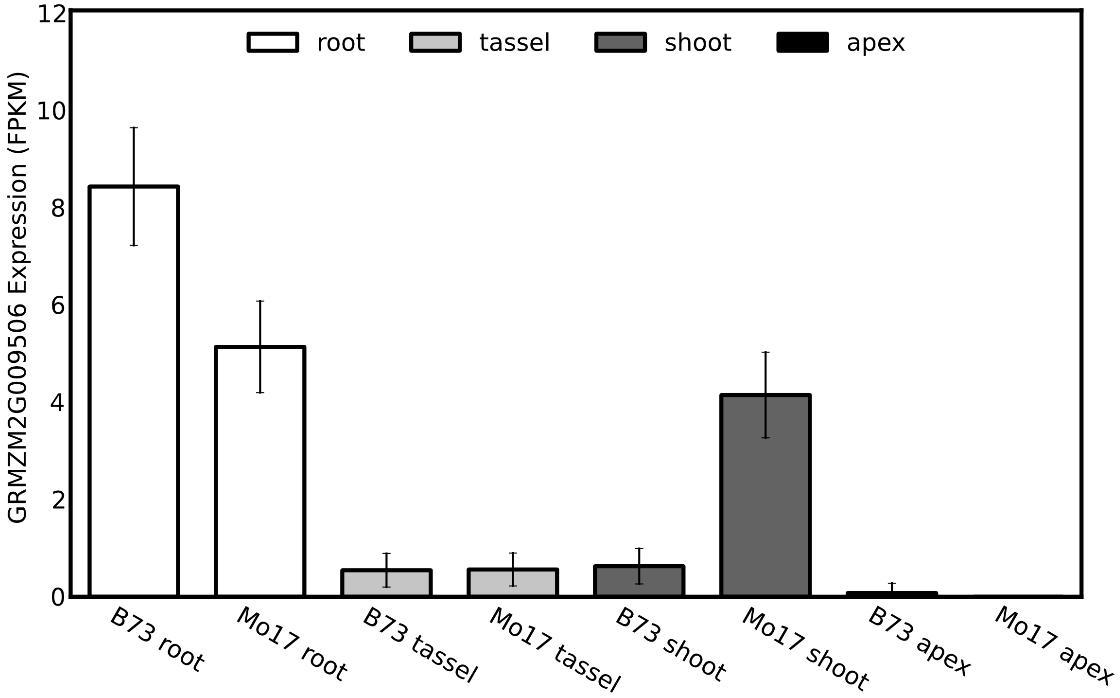

Supplemental Figure 5. Average high temperature data (http://www.noaa.gov) for four growing locations. The data is mean high temperature data from June 2009 to May 2012. Monthly temperature average weather station data from the National Oceanic and Atmospheric Administration (http://www.noaa.gov) was examined for locations closest to farm locations: Berkeley, CA (37.874, -122.260), Davis, CA (38.535, -121.776), and West Lafayette, IN (40.475, -86.992).

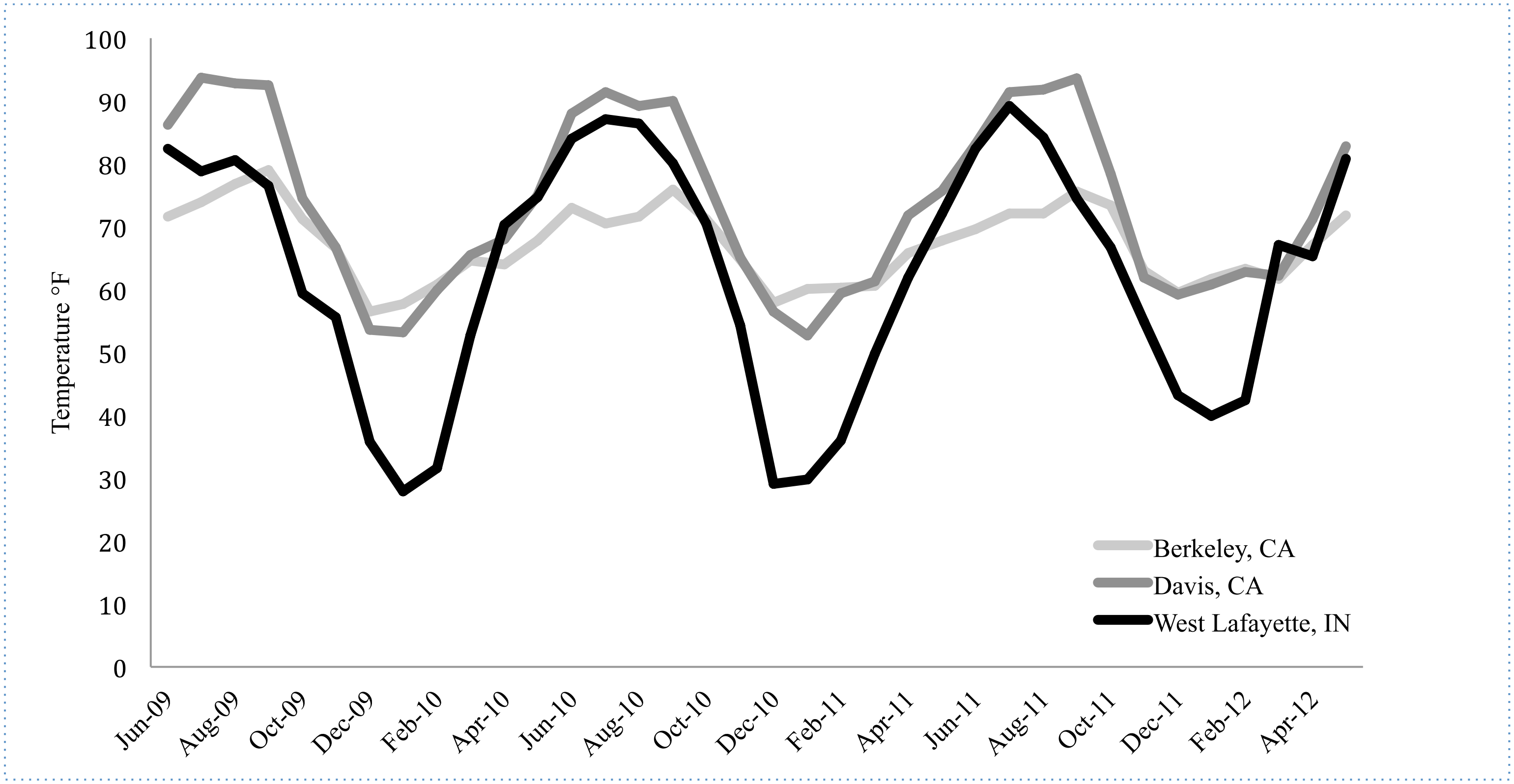

Supplemental Table 1. The genetic and physical positions of markers delimiting the *sol* QTL.

Marker	ISU Map position (cM)	AGP v1 (bp)	AGP v2 (bp)
umc2145	94.0	54,228,730 - 54,228,156	53,620,215- 54,536,375
umc1917	97.5	62,995,272 - 62,995,582	67,245,988 - 67,850,205
umc2227	99.7	66,028,648 - 66,027,760	67,777,456 - 67,778,133
bnl5.59a	133.5	183,652,591 - 183,652,422	183,804,477 - 183,817,286

Supplemental Table 2. Genotypes and marker positions of IBMs of intest examined near *sol* (umc2145) and *lcf* (nblg1247) genotypes based on the IBM ISU map version 4.

	Chr	Pos[1]	IBM 4[2]	IBM 18	IBM 25	IBM 30	IBM 47	IBM 55	IBM 65	IBM 69	IBM 72	B73[3]	Mo17[4]	Missing[5]	% B73
lim432[1]	1	91.1	B	B	B	B	B	A	B	B	B	1	8	0	0.1111
bnl12.06a	1	91.7	B	B	B	B	B	-	B	-	B	0	7	2	0.0000[M]
umc1598	1	92	B	B	B	B	B	A	B	B	B	1	8	0	0.1111
umc1880	1	92.4	B	B	B	-	B	A	B	B	B	1	7	1	0.1250
bnlg1866	1	92.4	B	B	-	B	B	A	B	B	B	1	7	1	0.1250
phi109275	1	92.4	B	B	B	B	B	A	-	-	B	1	6	2	0.1429
ndp1	1	92.4	B	B	B	B	B	A	B	B	-	1	7	1	0.1250
mmp151a	1	92.9	B	B	B	B	B	A	B	B	B	1	8	0	0.1111
cdo938a	1	93	B	B	B	B	B	A	B	-	B	1	7	1	0.1250
mmp23	1	93.4	B	B	B	B	B	A	B	B	B	1	8	0	0.1111
mmp23	1	93.4	B	B	B	B	B	A	B	B	B	1	8	0	0.1111
mmp56	1	93.9	B	B	B	B	B	A	B	-	B	1	7	1	0.1250
bnlg2238	1	94	B	B	B	B	B	A	B	B	B	1	8	0	0.1111
umc2124a	1	94	B	B	B	B	B	A	B	B	B	1	8	0	0.1111
umc2145	1	94	B	-	-	B	B	A	B	-	B	1	5	3	0.1667
IDP1489	1	94.5	B	-	A	B	B	B	B	B	B	1	7	1	0.1250
mmp100	1	95.2	B	B	B	B	B	A	B	B	B	1	8	0	0.1111
AY110393	1	95.7	B	A	B	B	B	A	B	B	-	2	6	1	0.2500
AY110393	1	95.7	B	B	B	B	B	A	B	B	B	1	8	0	0.1111
IDP739	1	96.6	B	-	A	B	B	B	B	B	B	1	7	1	0.1250
asg30b	1	96.6	B	B	B	-	B	B	B	B	B	0	8	1	0.0000[M]
IDP197	1	96.9	B	-	A	B	B	B	B	B	B	1	7	1	0.1250

umc2217	1	96.9	B	-	A	B	B	B	B	B	B	1	7	1	0.1250
umc1849	1	97	B	B	B	B	B	B	B	B	B	0	9	0	0.0000[M]
IDP585	1	97.5	B	-	A	B	B	B	B	B	B	1	7	1	0.1250
IDP182	1	97.5	B	-	A	B	B	B	B	B	B	1	7	1	0.1250
umc1917	1	97.5	B	B	B	B	B	B	B	B	B	0	9	0	0.0000[M]
AY110330	1	98.1	B	B	B	B	B	B	B	B	B	0	9	0	0.0000[M]
magi23951	1	98.4	B	-	A	B	B	B	B	B	B	1	7	1	0.1250
isu041b	1	98.4	B	B	B	B	B	B	-	-	B	0	7	2	0.0000[M]
ufg77	1	98.8	B	B	B	B	B	B	B	B	B	0	9	0	0.0000[M]
uaz248a(his3)	1	99.4	B	B	B	B	B	B	B	B	B	0	9	0	0.0000[M]
umc2227	1	99.7	B	B	B	B	B	B	B	B	B	0	9	0	0.0000[M]
IDP3943	1	100	B	-	A	B	B	B	B	B	B	1	7	1	0.1250
ufg43	1	101	B	B	B	B	B	B	B	B	B	0	9	0	0.0000[M]
IDP2553	1	101.6	B	-	B	B	B	B	B	B	B	0	8	1	0.1111
bnlg1811	1	102.3	B	B	B	B	B	B	B	B	B	0	9	0	0.0000[M]
IDP511	1	102.6	B	-	A	B	B	B	B	B	B	1	7	1	0.1250
AI855190	1	120.7	B	B	B	A	A	B	B	B	B	2	7	0	0.2222
mmp124	1	120.7	B	B	B	A	A	B	B	B	B	2	7	0	0.2222
AY110396	1	121.1	B	B	B	B	B	B	B	B	B	0	9	0	0
umc1906	1	121.1	B	B	B	A	-	B	B	B	B	1	7	1	0.1250
umc1601	1	121.6	B	B	B	A	A	B	B	B	B	2	7	0	0.2222
IDP311	7	65.7	A	-	A	A	A	A	A	A	A	8	0	1	0.8889
bnlg2203	7	66	A	A	A	A	A	A	A	A	A	9	0	0	1.0000[B]
bnlg1792	7	66	A	A	A	A	A	A	A	A	-	8	0	1	1.0000[B]
IDP3994	7	66	A	-	A	A	A	A	A	A	A	8	0	1	1.0000[B]

IDP2524	7	66	A	-	A	A	A	A	A	A	A	8	0	1	1.0000[B]
rz698d(ppy)	7	66.3	A	A	A	A	A	A	A	A	A	9	0	0	1.0000[B]
AY109809	7	66.3	A	A	A	A	A	A	A	A	A	9	0	0	1.0000[B]
bnlg1380	7	66.6	A	A	A	A	A	A	A	A	A	9	0	0	1.0000[B]
bnlg1247	7	66.6	A	A	A	A	A	A	A	A	A	9	0	0	1.0000[B]
IDP2464	7	66.6	A	-	A	A	A	A	A	A	A	8	0	1	1.0000[B]
psr371b	7	66.9	A	A	A	A	A	B	A	A	A	8	1	0	0.8889
cyp6	7	66.9	A	A	A	A	A	B	A	A	A	8	1	0	0.8889
cncr2	7	66.9	A	-	A	A	A	B	A	A	A	7	1	1	0.8750
IDP3810	7	66.9	A	-	A	A	A	B	A	A	A	7	1	1	0.8750
IDP837	7	66.9	A	-	A	A	A	B	A	A	A	7	1	1	0.8750
IDP767	7	66.9	A	-	B	A	A	B	A	A	A	6	2	1	0.7500
uaz187	7	66.9	A	A	A	A	A	B	A	A	A	8	1	0	0.8889
ufg121	7	66.9	A	A	A	A	A	B	A	A	A	8	1	0	0.8889
bnlg2233	7	67	A	A	-	A	A	A	A	A	A	8	0	1	1.0000[B]
mmp187	7	67.2	A	A	A	A	A	B	A	A	A	8	1	0	0.8889
bnlg1094	7	67.2	A	A	A	A	A	B	A	A	A	8	1	0	0.8889
rz698e(ppy)	7	67.2	A	A	A	A	A	A	A	A	A	9	0	0	1.0000[B]
IDP1643	7	67.2	A	-	A	A	A	B	A	A	A	7	1	1	0.8750
mmp26	7	67.2	A	A	A	A	A	B	A	A	A	8	1	0	0.8889
IDP84	7	67.5	A	-	A	A	A	B	A	A	A	7	1	1	0.8750
IDP3795	7	67.5	A	-	A	A	A	B	A	A	A	7	1	1	0.8750
IDP3971	7	67.8	A	-	A	A	A	B	A	A	A	7	1	1	0.8750
crt2	7	68.3	A	A	A	A	A	B	A	A	A	8	1	0	0.8889

crt2	7	68.3	A	A	A	A	A	B	A	A	A	8	1	0	0.8889
umc1932	7	68.6	A	A	A	A	A	A	A	A	A	9	0	0	1.0000[B]
umc1929	7	69	A	A	A	-	A	B	A	A	B	6	2	1	0.7500

[1] Marker position based on the ISU IBM Map version 4 (http://www.maizegdb.org).
[2] IBM RIL individuals with genotypes determined from the ISU IBM Map version 4 (http://www.maizegdb.org). The IBM RILs listed in this table previously showed rescued phenotype. B73 is indicated by an "A". Mo17 is represented by a "B". Missing data is represented by a "-".
[3] Indicates the number of B73 genotypes across the IBM RILs of interest listed in the table.
[4] Indicates the number of Mo17 genotypes across the IBM RILs of interest listed in the table.
[5] Indicates the number of missing genotypes summed across the IBM RILs of interest.
[B] Regions which contain mostly B73 across all IBM RILs of interest are indicated with a B.
[M] Regions which contain mostly Mo17 are indicated with an M.

Files S1-S2

Available for download at http://www.g3journal.org/lookup/suppl/doi:10.1534/g3.114.014183/-/DC1
