# Supplementary material for: Natural Variation at sympathy for the ligule Controls Penetrance of the Semidominant Liguleless narrow-R Mutation in Zea mays
Source: G3 (Bethesda). 2014 Oct 24;4(12):2297–306. doi: 10.1534/g3.114.014183 (PMC4267926; doi:10.1534/g3.114.014183)

Supplemental Figure 1. Leaf length and width measurements for five IBM RIL X *Lgn-R*/+ F1 individuals displaying near wild-type B73 phenotypes or rescued (indicated in blue) and five individuals displaying near *Lgn-R* phenotypes or suppressed (indicated in red). Measurements were made in triplicate in 3 locations: two independent plantings in West Lafayette, IN (Purdue University Agronomy Center for Research and Education) and Albany, CA (University of California Gill Tract Farm). The individuals indicated (IBM18, IBM30, IBM69 and IBM72) are IBM RIL X *Lgn-R*/+ F1 individuals that are phenotypically near wild-type Mo17 in leaf length and width.

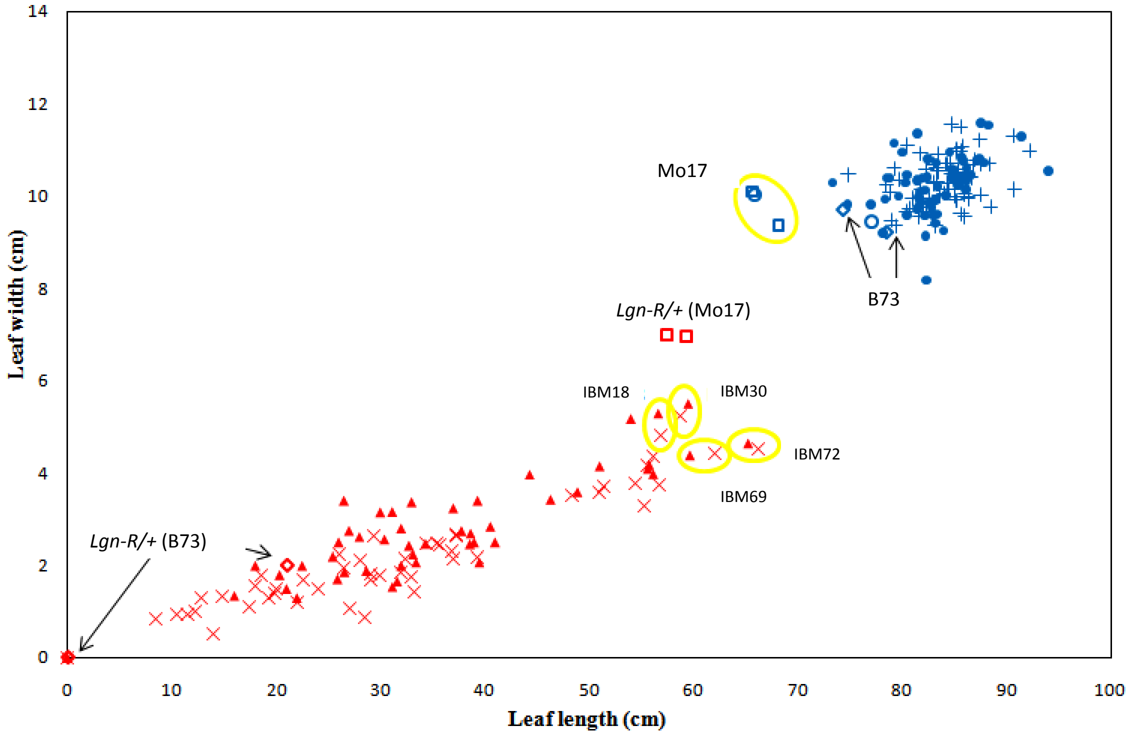

Supplement: Supporting Information [file supp_g3.114.014183_FigureS1.pdf]
