# Supplementary material for: Natural Variation at sympathy for the ligule Controls Penetrance of the Semidominant Liguleless narrow-R Mutation in Zea mays
Source: G3 (Bethesda). 2014 Oct 24;4(12):2297–306. doi: 10.1534/g3.114.014183 (PMC4267926; doi:10.1534/g3.114.014183)
Supplement: Supporting Information [file supp_g3.114.014183_FigureS2.pdf]

Supplemental Figure 2. Effect plots (R/qtl Broman et al 2003) for each trait comparing sol (umc2145) and lcf (nbp1) markers. The B73 allele (AA) and the Mo17 allele (BB) are indicated on the x-axis for lcf (nbp1). sol (umc2145) is represented by a red line for B73 and a blue line for Mo17.**A-C.** Effect plots for the count data for the **A.** GT location; **B.** IL1 location; **C.** IN 2 location. **D-F** are effect plots for the length and width data: **D.** PCA value for length and width, IN only; **E.** PCA value for the length and width measurements, GT only; **F.** PCA value for the length and width measurements, all locations. **G-H.** effect plots for the leaf area data: **G.** leaf area measurements for GT only; **H.** PCA value for IN.

S2A

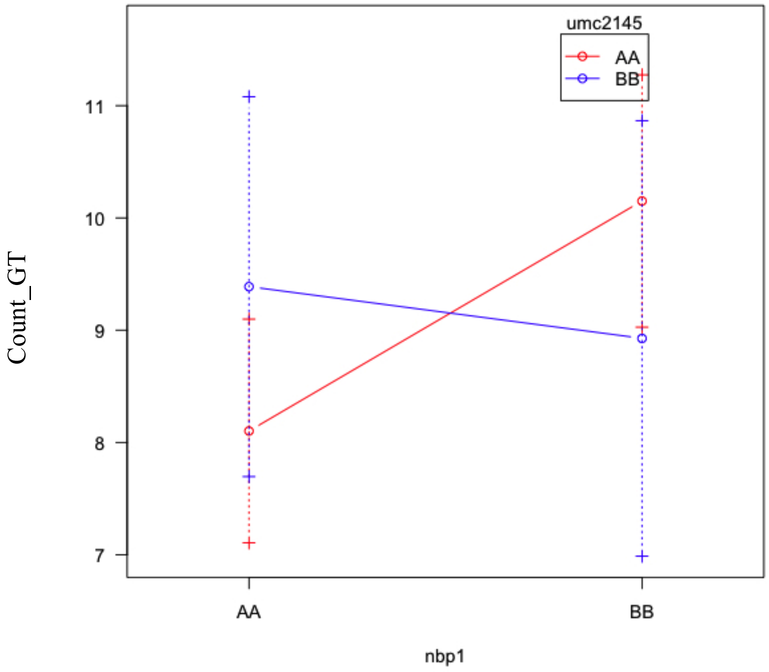

S2B

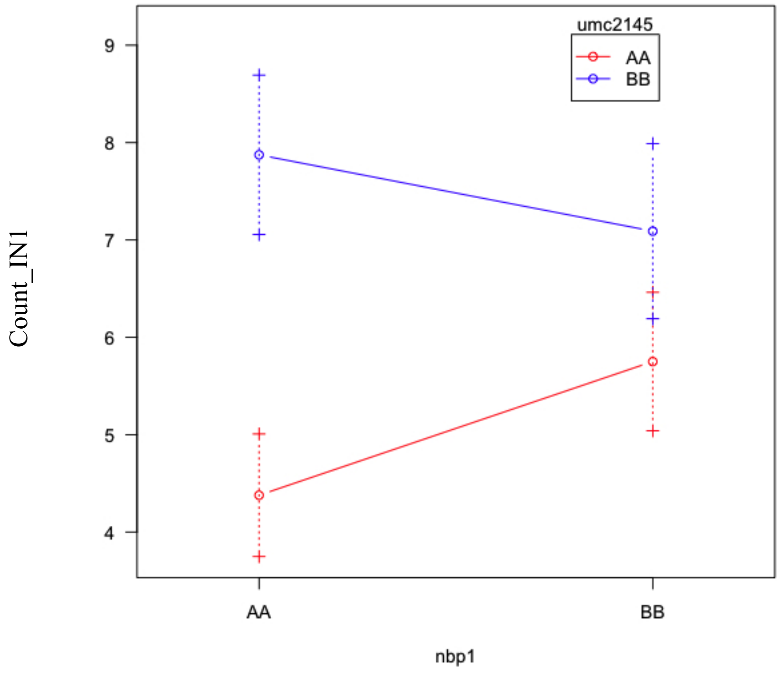

S2C

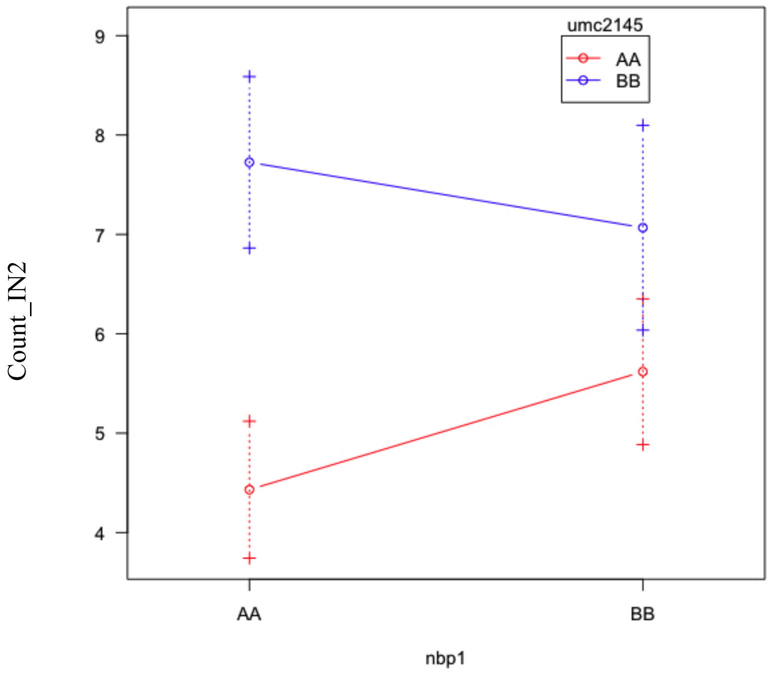
umc2145
AA
BB
9
8
7
6
5
4
Count_IN2
AA
BB
nbp1

S2D

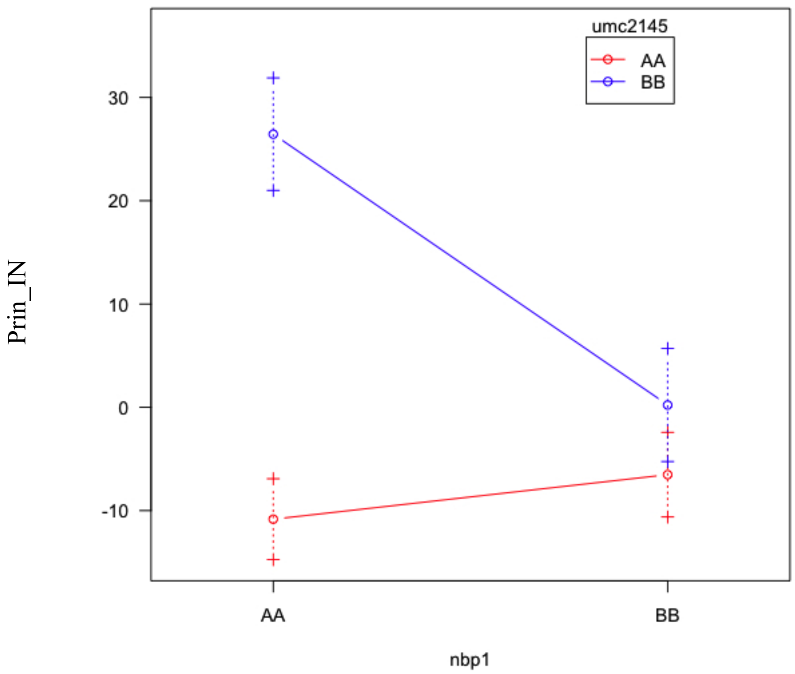
umc2145
AA
BB
30
20
10
0
-10
Prin_IN
AA
BB
nbp1

S2E

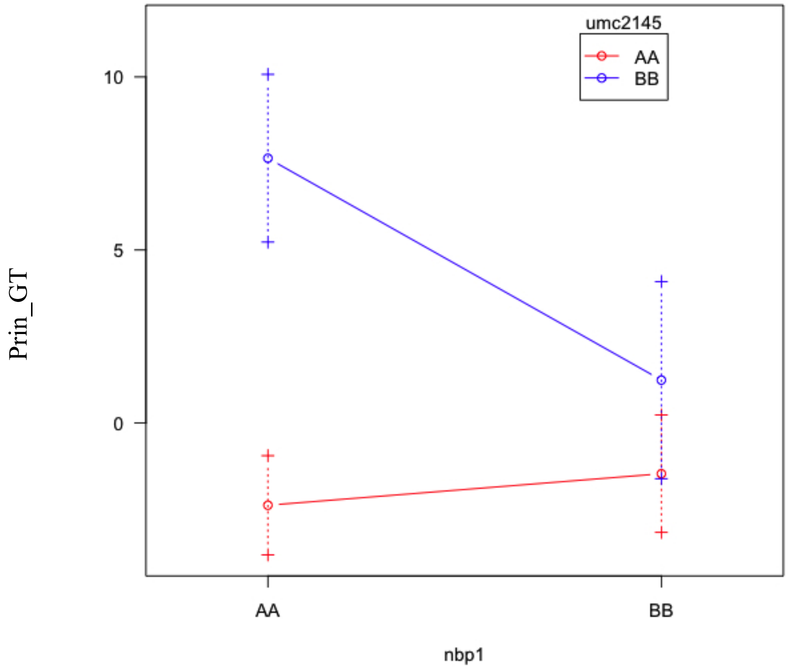
umc2145
AA
BB
10
5
0
Prin_GT
AA
BB
nbp1

S2F

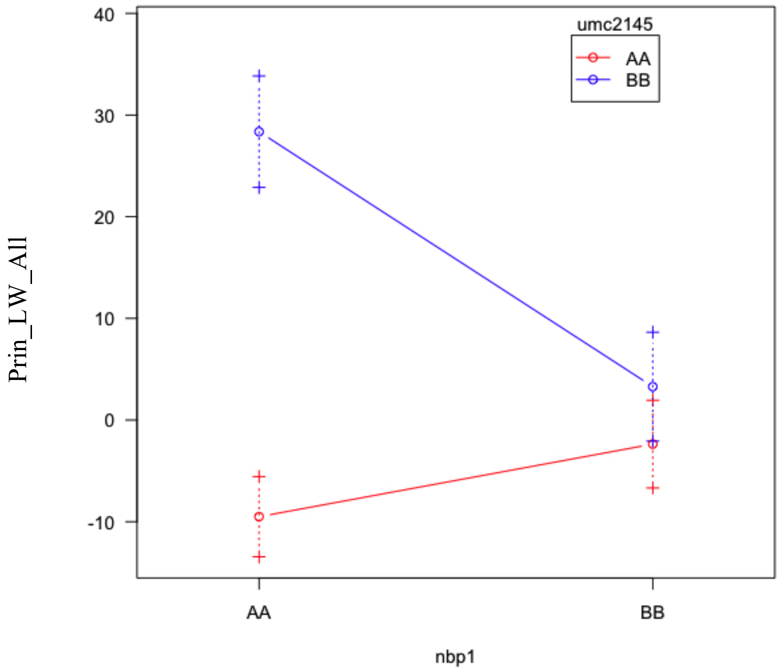
umc2145
AA
BB
40
30
20
10
0
-10
Prin_LW_All
AA
BB
nbp1

S2G

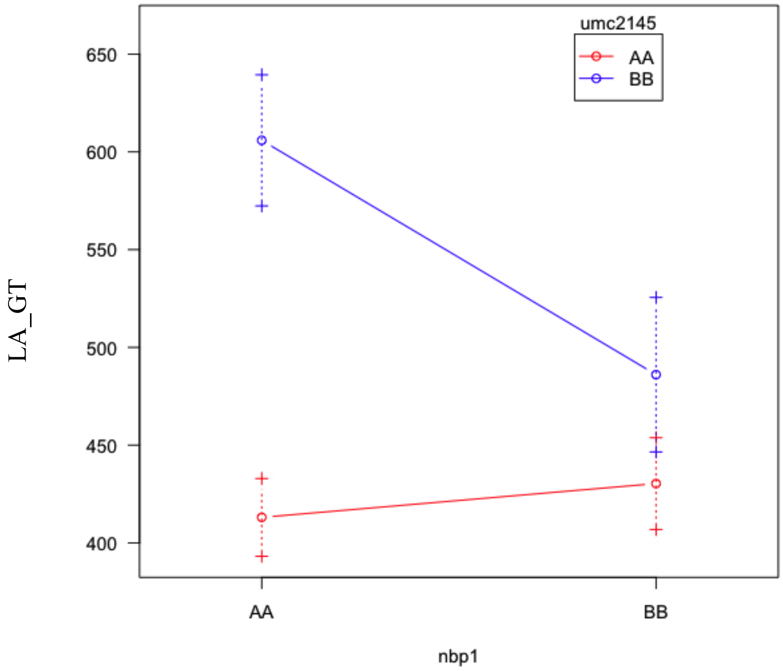
umc2145
AA
BB
650
600
550
500
450
400
LA_GT
AA
BB
nbp1

S2H

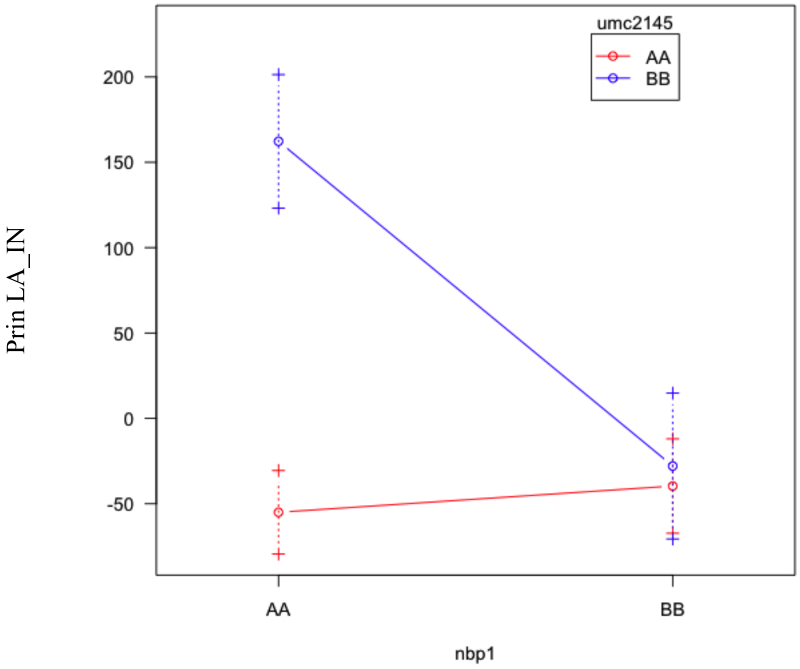
umc2145
AA
BB
200
150
100
50
0
-50
Prin LA_IN_
AA
BB
nbp1
