# Supplementary material for: Natural Variation at sympathy for the ligule Controls Penetrance of the Semidominant Liguleless narrow-R Mutation in Zea mays
Source: G3 (Bethesda). 2014 Oct 24;4(12):2297–306. doi: 10.1534/g3.114.014183 (PMC4267926; doi:10.1534/g3.114.014183)

Supplemental Figure 3. Histogram of RNAseq data describing mRNA accumulation for *Lgn* (GRMZM2G134382) in multiple tissue tyes for both B73 and Mo17. RNAseq data was generated using the qTeller tool (http://qteller.com/). Tissue types are as follows: root, shoot, tassel and shoot apex. The HTML link for regenerating this analysis at the qTeller website is:

http://qteller.com/NAM/bar_chart.php?name=GRMZM2G134382&info=B73_root%7CMo17_root%7CMo17_sam_apex%7CB73_sam_apex%7CMo17_tassel%7CB73_tassel%7CMo17_shoot%7CB73_shoot

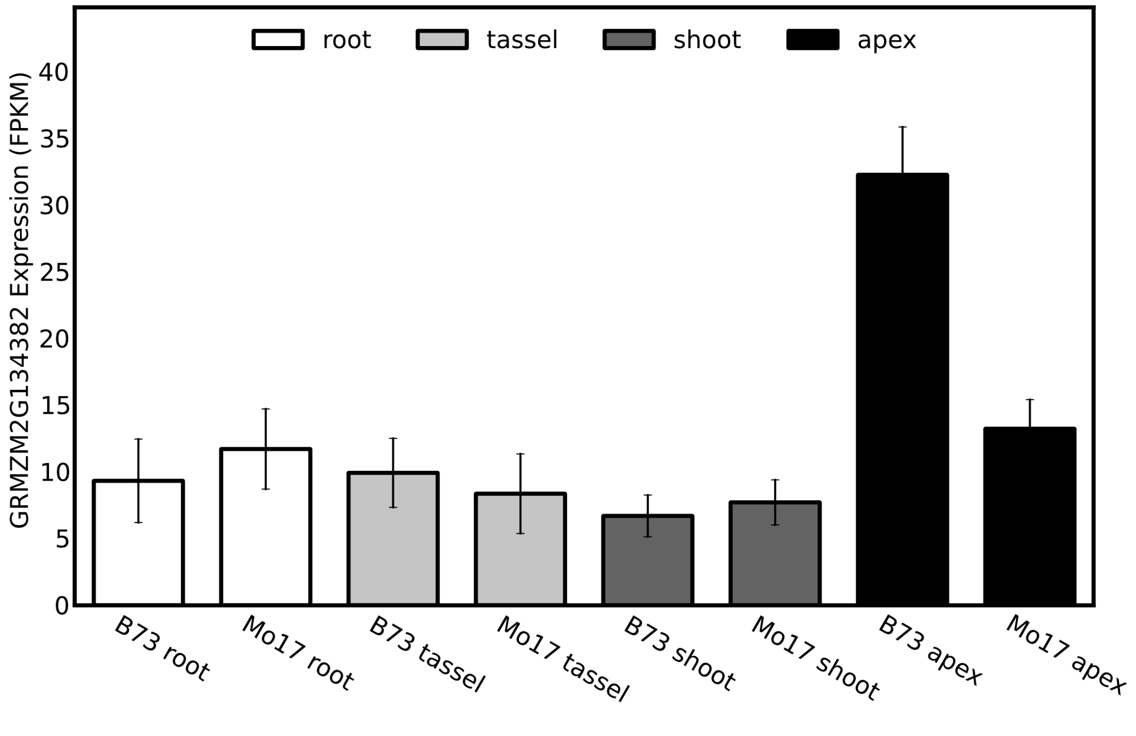

Supplement: Supporting Information [file supp_g3.114.014183_FigureS3.pdf]
