# Supplementary material for: Natural Variation at sympathy for the ligule Controls Penetrance of the Semidominant Liguleless narrow-R Mutation in Zea mays
Source: G3 (Bethesda). 2014 Oct 24;4(12):2297–306. doi: 10.1534/g3.114.014183 (PMC4267926; doi:10.1534/g3.114.014183)

Supplemental Figure 4. RNAseq expression data for *Sln* (GRMZM2G009506) in B73 and Mo17 across multiple tissue types. mRNA data was generated using qTeller (http://qteller.com/). Tissue types are as follows: root, shoot, tassel and shoot apex. The HTML link for regenerating this analysis at the qTeller website is: http://qteller.com/NAM/bar_chart.php?name=GRMZM2G009506&info=B73_root%7CMo17_root%7CMo17_sam_apex%7CB73_sam_apex%7CMo17_tassel%7CB73_tassel%7CMo17_shoot%7CB73_shoot

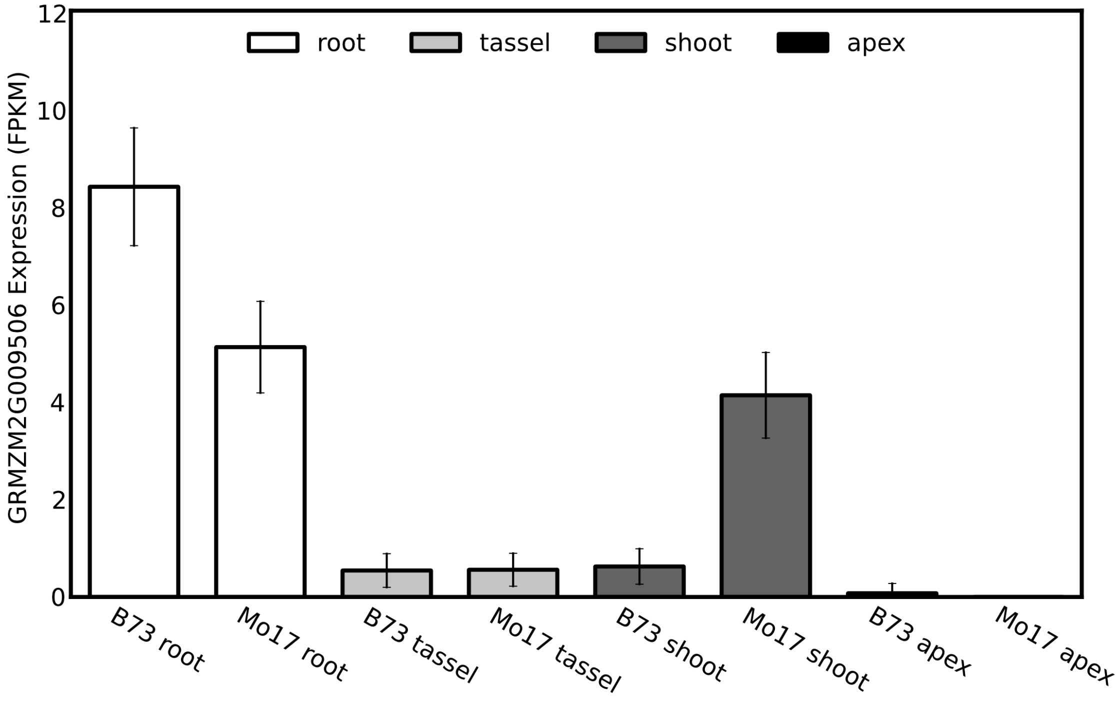

Supplement: Supporting Information [file supp_g3.114.014183_FigureS4.pdf]
