# Supplementary material for: Natural Variation at sympathy for the ligule Controls Penetrance of the Semidominant Liguleless narrow-R Mutation in Zea mays
Source: G3 (Bethesda). 2014 Oct 24;4(12):2297–306. doi: 10.1534/g3.114.014183 (PMC4267926; doi:10.1534/g3.114.014183)

Supplemental Figure 5. Average high temperature data (http://www.noaa.gov) for four growing locations. The data is mean high temperature data from June 2009 to May 2012. Monthly temperature average weather station data from the National Oceanic and Atmospheric Administration (http://www.noaa.gov) was examined for locations closest to farm locations: Berkeley, CA (37.874, -122.260), Davis, CA (38.535, -121.776), and West Lafayette, IN (40.475, -86.992).

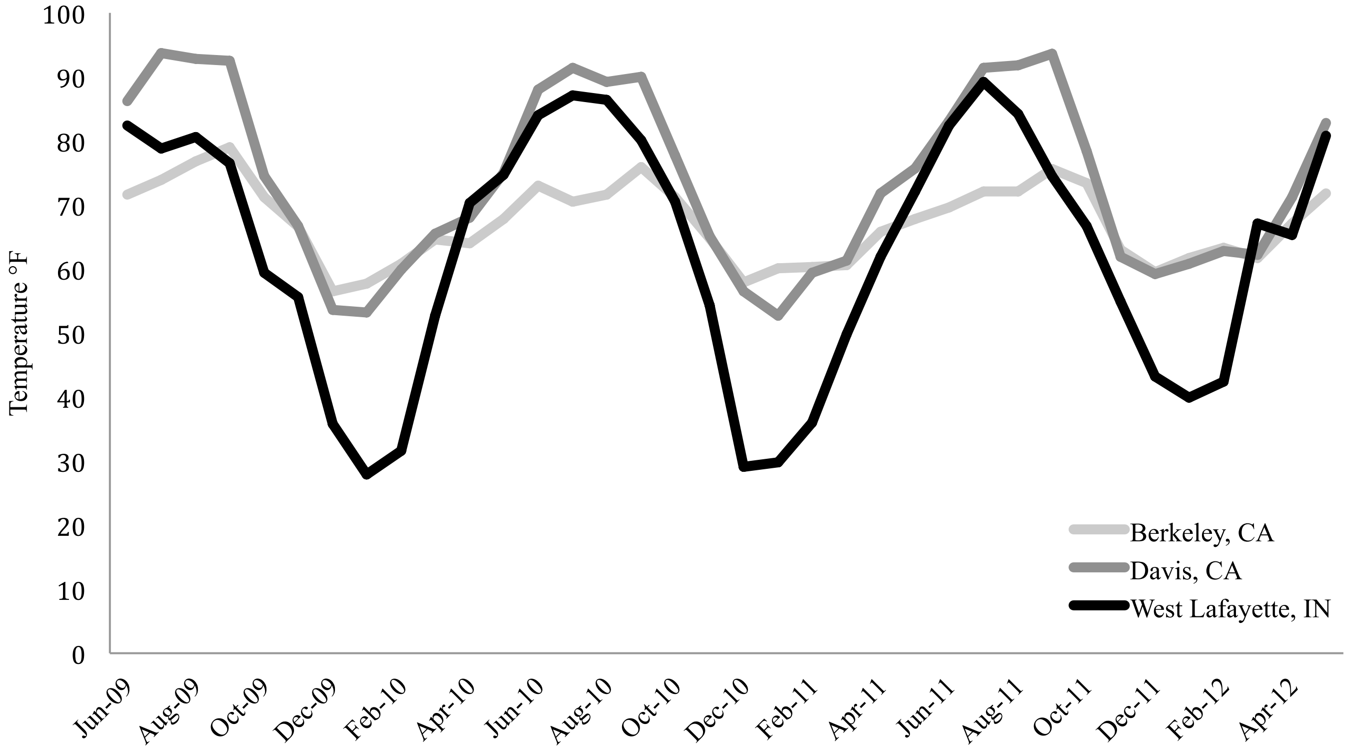

Supplement: Supporting Information [file supp_g3.114.014183_FigureS5.pdf]
