# Supplementary material for: Natural Variation at sympathy for the ligule Controls Penetrance of the Semidominant Liguleless narrow-R Mutation in Zea mays
Source: G3 (Bethesda). 2014 Oct 24;4(12):2297–306. doi: 10.1534/g3.114.014183 (PMC4267926; doi:10.1534/g3.114.014183)
Supplement: Supporting Information [file supp_g3.114.014183_TableS1.pdf]

Supplemental Table 1. The genetic and physical positions of markers delimiting the *sol* QTL.

Marker	ISU Map position (cM)	AGP v1 (bp)	AGP v2 (bp)
umc2145	94.0	54,228,730 - 54,228,156	53,620,215- 54,536,375
umc1917	97.5	62,995,272 - 62,995,582	67,245,988 - 67,850,205
umc2227	99.7	66,028,648 - 66,027,760	67,777,456 - 67,778,133
bnl5.59a	133.5	183,652,591 - 183,652,422	183,804,477 - 183,817,286
