# Supplementary material for: Natural Variation at sympathy for the ligule Controls Penetrance of the Semidominant Liguleless narrow-R Mutation in Zea mays
Source: G3 (Bethesda). 2014 Oct 24;4(12):2297–306. doi: 10.1534/g3.114.014183 (PMC4267926; doi:10.1534/g3.114.014183)
Supplement: Supporting Information [file supp_g3.114.014183_TableS2.pdf]

Supplemental Table 2. Genotypes and marker positions of IBMs of intest examined near *sol* (umc2145) and *lcf* (nblg1247) genotypes based on the IBM ISU map version 4.

	Chr	Pos[1]	IBM 4[2]	IBM 18	IBM 25	IBM 30	IBM 47	IBM 55	IBM 65	IBM 69	IBM 72	B73[3]	Mo17[4]	Missing[5]	% B73
lim432[1]	1	91.1	B	B	B	B	B	A	B	B	B	1	8	0	0.1111
bnl12.06a	1	91.7	B	B	B	B	B	-	B	-	B	0	7	2	0.0000[M]
umc1598	1	92	B	B	B	B	B	A	B	B	B	1	8	0	0.1111
umc1880	1	92.4	B	B	B	-	B	A	B	B	B	1	7	1	0.1250
bnlg1866	1	92.4	B	B	-	B	B	A	B	B	B	1	7	1	0.1250
phi109275	1	92.4	B	B	B	B	B	A	-	-	B	1	6	2	0.1429
ndp1	1	92.4	B	B	B	B	B	A	B	B	-	1	7	1	0.1250
mmp151a	1	92.9	B	B	B	B	B	A	B	B	B	1	8	0	0.1111
cdo938a	1	93	B	B	B	B	B	A	B	-	B	1	7	1	0.1250
mmp23	1	93.4	B	B	B	B	B	A	B	B	B	1	8	0	0.1111
mmp23	1	93.4	B	B	B	B	B	A	B	B	B	1	8	0	0.1111
mmp56	1	93.9	B	B	B	B	B	A	B	-	B	1	7	1	0.1250
bnlg2238	1	94	B	B	B	B	B	A	B	B	B	1	8	0	0.1111
umc2124a	1	94	B	B	B	B	B	A	B	B	B	1	8	0	0.1111
umc2145	1	94	B	-	-	B	B	A	B	-	B	1	5	3	0.1667
IDP1489	1	94.5	B	-	A	B	B	B	B	B	B	1	7	1	0.1250
mmp100	1	95.2	B	B	B	B	B	A	B	B	B	1	8	0	0.1111
AY110393	1	95.7	B	A	B	B	B	A	B	B	-	2	6	1	0.2500
AY110393	1	95.7	B	B	B	B	B	A	B	B	B	1	8	0	0.1111
IDP739	1	96.6	B	-	A	B	B	B	B	B	B	1	7	1	0.1250
asg30b	1	96.6	B	B	B	-	B	B	B	B	B	0	8	1	0.0000[M]
IDP197	1	96.9	B	-	A	B	B	B	B	B	B	1	7	1	0.1250

umc2217	1	96.9	B	-	A	B	B	B	B	B	B	1	7	1	0.1250
umc1849	1	97	B	B	B	B	B	B	B	B	B	0	9	0	0.0000[M]
IDP585	1	97.5	B	-	A	B	B	B	B	B	B	1	7	1	0.1250
IDP182	1	97.5	B	-	A	B	B	B	B	B	B	1	7	1	0.1250
umc1917	1	97.5	B	B	B	B	B	B	B	B	B	0	9	0	0.0000[M]
AY110330	1	98.1	B	B	B	B	B	B	B	B	B	0	9	0	0.0000[M]
magi23951	1	98.4	B	-	A	B	B	B	B	B	B	1	7	1	0.1250
isu041b	1	98.4	B	B	B	B	B	B	-	-	B	0	7	2	0.0000[M]
ufg77	1	98.8	B	B	B	B	B	B	B	B	B	0	9	0	0.0000[M]
uaz248a(his3)	1	99.4	B	B	B	B	B	B	B	B	B	0	9	0	0.0000[M]
umc2227	1	99.7	B	B	B	B	B	B	B	B	B	0	9	0	0.0000[M]
IDP3943	1	100	B	-	A	B	B	B	B	B	B	1	7	1	0.1250
ufg43	1	101	B	B	B	B	B	B	B	B	B	0	9	0	0.0000[M]
IDP2553	1	101.6	B	-	B	B	B	B	B	B	B	0	8	1	0.1111
bnlg1811	1	102.3	B	B	B	B	B	B	B	B	B	0	9	0	0.0000[M]
IDP511	1	102.6	B	-	A	B	B	B	B	B	B	1	7	1	0.1250
AI855190	1	120.7	B	B	B	A	A	B	B	B	B	2	7	0	0.2222
mmp124	1	120.7	B	B	B	A	A	B	B	B	B	2	7	0	0.2222
AY110396	1	121.1	B	B	B	B	B	B	B	B	B	0	9	0	0
umc1906	1	121.1	B	B	B	A	-	B	B	B	B	1	7	1	0.1250
umc1601	1	121.6	B	B	B	A	A	B	B	B	B	2	7	0	0.2222
IDP311	7	65.7	A	-	A	A	A	A	A	A	A	8	0	1	0.8889
bnlg2203	7	66	A	A	A	A	A	A	A	A	A	9	0	0	1.0000[B]
bnlg1792	7	66	A	A	A	A	A	A	A	A	-	8	0	1	1.0000[B]
IDP3994	7	66	A	-	A	A	A	A	A	A	A	8	0	1	1.0000[B]

IDP2524	7	66	A	-	A	A	A	A	A	A	A	8	0	1	1.0000[B]
rz698d(ppy)	7	66.3	A	A	A	A	A	A	A	A	A	9	0	0	1.0000[B]
AY109809	7	66.3	A	A	A	A	A	A	A	A	A	9	0	0	1.0000[B]
bnlg1380	7	66.6	A	A	A	A	A	A	A	A	A	9	0	0	1.0000[B]
bnlg1247	7	66.6	A	A	A	A	A	A	A	A	A	9	0	0	1.0000[B]
IDP2464	7	66.6	A	-	A	A	A	A	A	A	A	8	0	1	1.0000[B]
psr371b	7	66.9	A	A	A	A	A	B	A	A	A	8	1	0	0.8889
cyp6	7	66.9	A	A	A	A	A	B	A	A	A	8	1	0	0.8889
cncr2	7	66.9	A	-	A	A	A	B	A	A	A	7	1	1	0.8750
IDP3810	7	66.9	A	-	A	A	A	B	A	A	A	7	1	1	0.8750
IDP837	7	66.9	A	-	A	A	A	B	A	A	A	7	1	1	0.8750
IDP767	7	66.9	A	-	B	A	A	B	A	A	A	6	2	1	0.7500
uaz187	7	66.9	A	A	A	A	A	B	A	A	A	8	1	0	0.8889
ufg121	7	66.9	A	A	A	A	A	B	A	A	A	8	1	0	0.8889
bnlg2233	7	67	A	A	-	A	A	A	A	A	A	8	0	1	1.0000[B]
mmp187	7	67.2	A	A	A	A	A	B	A	A	A	8	1	0	0.8889
bnlg1094	7	67.2	A	A	A	A	A	B	A	A	A	8	1	0	0.8889
rz698e(ppy)	7	67.2	A	A	A	A	A	A	A	A	A	9	0	0	1.0000[B]
IDP1643	7	67.2	A	-	A	A	A	B	A	A	A	7	1	1	0.8750
mmp26	7	67.2	A	A	A	A	A	B	A	A	A	8	1	0	0.8889
IDP84	7	67.5	A	-	A	A	A	B	A	A	A	7	1	1	0.8750
IDP3795	7	67.5	A	-	A	A	A	B	A	A	A	7	1	1	0.8750
IDP3971	7	67.8	A	-	A	A	A	B	A	A	A	7	1	1	0.8750
crt2	7	68.3	A	A	A	A	A	B	A	A	A	8	1	0	0.8889

crt2	7	68.3	A	A	A	A	A	B	A	A	A	8	1	0	0.8889
umc1932	7	68.6	A	A	A	A	A	A	A	A	A	9	0	0	1.0000[B]
umc1929	7	69	A	A	A	-	A	B	A	A	B	6	2	1	0.7500

[1] Marker position based on the ISU IBM Map version 4 (http://www.maizegdb.org).
[2] IBM RIL individuals with genotypes determined from the ISU IBM Map version 4 (http://www.maizegdb.org). The IBM RILs listed in this table previously showed rescued phenotype. B73 is indicated by an "A". Mo17 is represented by a "B". Missing data is represented by a "-".
[3] Indicates the number of B73 genotypes across the IBM RILs of interest listed in the table.
[4] Indicates the number of Mo17 genotypes across the IBM RILs of interest listed in the table.
[5] Indicates the number of missing genotypes summed across the IBM RILs of interest.
[B] Regions which contain mostly B73 across all IBM RILs of interest are indicated with a B.
[M] Regions which contain mostly Mo17 are indicated with an M.
